# Investigation of tumor-tumor interactions in a double human cervical carcinoma xenograft model in nude mice

**DOI:** 10.18632/oncotarget.25140

**Published:** 2018-04-24

**Authors:** Barbara Mertens, Tatiane Cristina de Araujo Nogueira, Dimitrios Topalis, Ruzena Stranska, Robert Snoeck, Graciela Andrei

**Affiliations:** ^1^ Rega Institute for Medical Research, KU Leuven, Leuven, Belgium

**Keywords:** tumor-tumor interactions, SiHa cervical carcinoma, double xenograft model, cidofovir, far-reaching antitumor effects

## Abstract

Tumor-tumor distant interactions within one organism are of major clinical relevance determining clinical outcome. To investigate this poorly understood phenomenon, a double human cervical xenograft model in nude mice was developed. A first tumor was induced subcutaneously by injection of human papillomavirus positive cervical carcinoma cells into the mouse lower right flank and 3 weeks later, animals were challenged with the same tumor cell line injected subcutaneously into the upper left flank. These tumors had no direct physical contact and we found no systemic changes induced by the primary tumor affecting the growth of a secondary tumor. However, ablation of the primary tumor by local treatment with cidofovir, a nucleotide analogue with known antiviral and antiproliferative properties, resulted not only in a local antitumor effect but also in a temporary far-reaching effect leading to retarded growth of the challenged tumor. Cidofovir far-reaching effects were linked to a reduced tumor-driven inflammation, to increased anti-tumor immune responses, and could not be enhanced by co-administration with immune stimulating adjuvants. Our findings point to the potential use of cidofovir in novel therapeutic strategies aiming to kill tumor cells as well as to influence the immune system to fight cancer.

## INTRODUCTION

Tumor interactions within a single organism have major clinical implications, mainly in the context of surgery and metastatic disease [1]. An increasing amount of experimental evidence which indicates that certain tumors can affect the behavior of other tumor(s) residing at different anatomical sites is available. However, the mechanisms underlying these systemic tumor interactions within a host remain poorly understood [1]. At present, it is accepted that tumor derived factors are capable of governing tumor progression at distant sites [3]. Both the concepts of systemic instigation and systemic inhibition have been described, although these mechanisms are not necessarily mutually exclusive.

Certain human tumors (named instigators), first described in breast cancers, were shown to facilitate the growth of otherwise indolent tumor cells (termed responders) residing at distant anatomical sites in a nude mice xenograft model [4-6]. This at-a-distance process, termed systemic instigation, was largely associated with the ability of instigating breast carcinoma cells to alter the host bone marrow, resulting in the generation of cells that, following mobilization into the general circulation, facilitate the growth of responding breast carcinomas [2, 4]. Tumor instigation could provide an explanation to some of the observations made in clinical oncology, such as differences in metastatic behavior of breast cancer subtypes, including differences in risk, timing and site of metastasis [5, 7, 8]. This phenomenon may explain why patients with one malignant neoplasm have an increased risk of developing independent primary cancers within a relatively short time after initial diagnosis [7, 9]. In addition, it may explain the observation that surgical removal of a primary breast tumor resulted in improved survival in women presenting distant breast metastasis at the time of primary diagnosis [10, 11].

On the other hand, concomitant tumor resistance, characterized by a controlling action of a tumor on the appearance and growth of another tumor, has been described in animal models [12, 13] as well as in clinical case reports [14, 15]. In syngeneic mice with two simultaneous implanted tumors, it was shown that only the growth of one of the tumors was significantly reduced [1]. Thus, in C57BL/6 mice subcutaneously injected with murine Lewis lung carcinoma cells on the two lateral sides of the caudal half of the back, one of the tumors had an exponential growth while the development of the other one was suppressed. Surgery, the mainstay treatment of most solid tumors, may be curative in the absence of dissemination of malignant cells from the primary tumor and is recommended in most clinical situations. However, surgical removal of a primary tumor may release the inhibitory pressure of a tumor on occult secondary sites leading to post-surgery metastatic acceleration, a phenomenon that can be explained by concomitant resistance [12, 13]. Some therapeutic options, such as preoperative administration of chemotherapy or radiotherapy, antioxidant agents and immunotherapy have been proposed to overcome metastatic growth after tumor removal [1, 13]. However, it is also claimed that *in situ* tumor destruction (ablation) can mediate antigen specific cellular immunity via presentation of processed antigens [16]. Furthermore, local photodynamic therapy of rat C6 glioma xenografts resulted in eradication of the primary tumor and reduced lung metastasis [17]. Activation of local and systemic antitumor immune responses by ablation of solid tumors with intratumoral electrochemical or alpha radiation treatments inhibited both breast and colon primary tumor growth, reduced the lung metastasis and prolonged animal survival in mice [16]. The destruction of the tumor, stimulated by these ablative treatments, could be further augmented in combination with an immune adjuvant.

Cervical cancer is the second most common malignancy affecting women worldwide [18]. This cancer is principally linked to a persistent infection with a high-risk human papillomavirus (HPV) type, mostly HPV-16 and HPV-18 [18-20]. The incidence rates of new primary cancers are higher among survivors of cervical cancer in comparison with the general population [21-23]. This has been ascribed to the presence of established risk factors in these patients, including high tobacco and/or alcohol consumption, hormonal and nutritional factors, exposure to the virus (HPV), genetic predisposition, late adverse effects of first cancer treatments and interactions among these factors [21]. To date, systemic tumor interactions in cervical cancer have not been investigated.

To evaluate the influence of a cervical cancer tumor on the development and growth of another tumor, we used a double xenograft model in nude mice. In this model, a first tumor xenograft was induced subcutaneously (s.c.) by injection of the HPV-16 cervical carcinoma SiHa cell line into one anatomical site (right flank) and later on, animals were challenged with tumor cells injected subcutaneously into a distant anatomical site (contralateral flank). These tumors had no direct physical contact, allowing for the study of systemic changes induced by the primary tumor on the growth of a secondary tumor. We also investigated whether local treatment with cidofovir (CDV), a nucleotide analogue with known antiviral and antiproliferative properties [24-27], would not only have a local antitumor effect but also a far-reaching (FR) effect leading to retarded growth of a challenged tumor. This nucleotide analogue was previously demonstrated to have antiproliferative effects *in vitro* and to improve the pathology caused by the growth of HPV^+^ cervical carcinoma xenografts [28] as well as of other tumor xenografts in athymic nude mice [29-31]. To enhance the FR effects induced by cidofovir, we investigated the use of apoptotic tumor cells as a source of a wide variety of tumor antigens able to induce a more integral immune response, and co-administration of cidofovir together with immune stimulating agents.

## RESULTS

### The presence of a primary cervical carcinoma xenograft had no impact on the growth of a secondary tumor xenograft induced at a distant anatomical site

**Figure 1:**
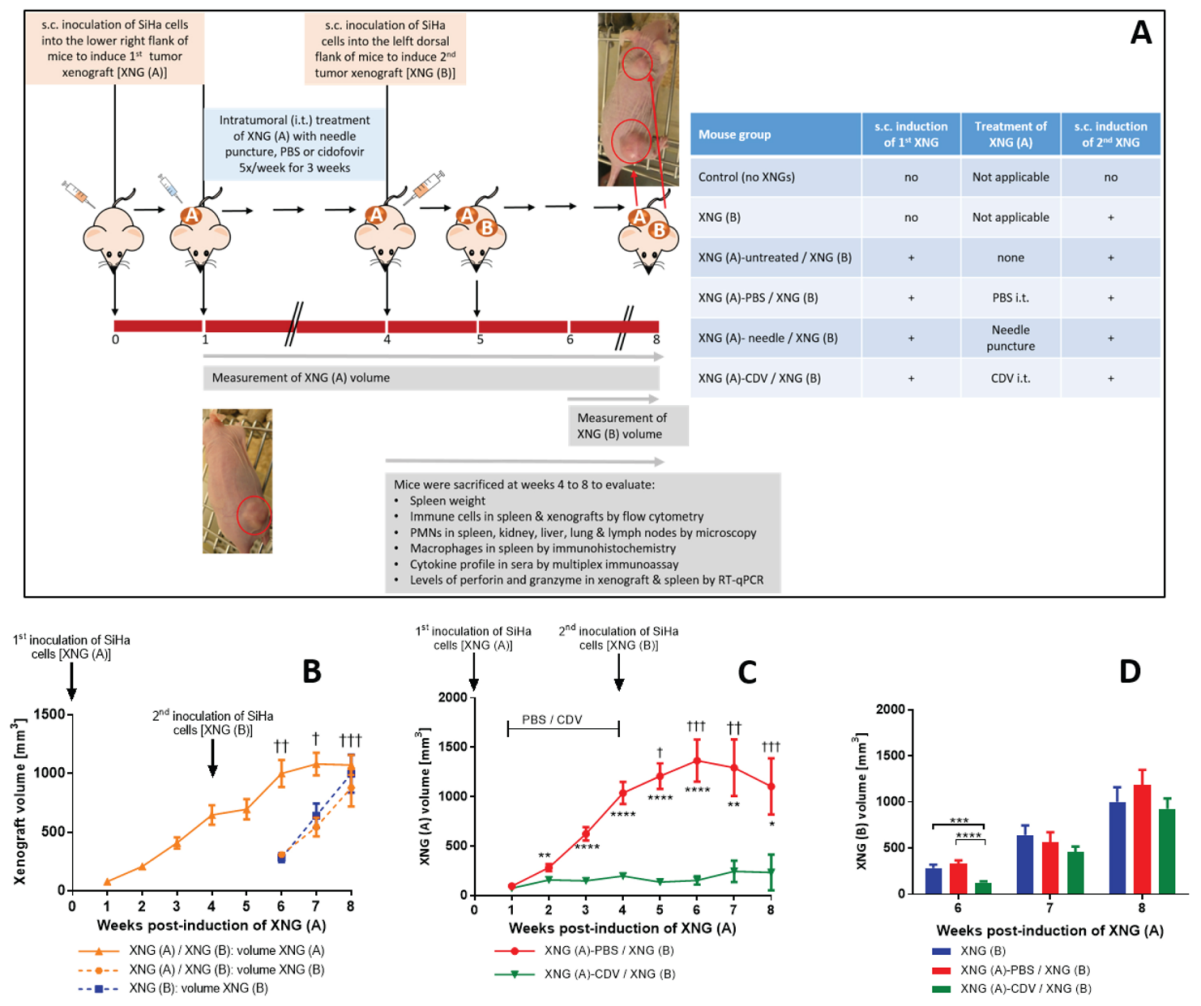
Tumor growth in a double subcutaneous SiHa cells xenograft mouse model **(A)** Mice were inoculated subcutaneously (s.c.) into the lower right flank with 2×10^6^ SiHa cells in 200 μl PBS [primary xenograft, XNG (A)]. Intratumoral (i.t.) treatment with needle puncture, PBS or cidofovir started one week after injection of the SiHa cells and was performed 5 times per week for 3 weeks. Four weeks after injection of XNG (A), a second xenograft [XNG (B)] was injected (2×10^6^ SiHa cells in 200 μl PBS) into the left dorsal flank. From week 4 to 8, mice were euthanized for evaluation of different disease parameters. **(B)** Volume of XNG (A) and XNG (B) in animals bearing two xenografts [XNG (A) / XNG (B)] and animals having been induced only a xenograft in the left dorsal flank [XNG (B)]. Volume of XNG (A) **(C)** and XNG (B) **(D)** in mice that had the primary xenograft treated with PBS [XNG (A)-PBS / XNG (B)] or cidofovir [XNG (A)-CDV / XNG (B)]. Xenograft volume was measured once per week from week 1 [XNG (A)] or week 6 [XNG (B)] onwards. Mice that died or had to be euthanized for ethical reasons are indicated on the graph by a cross. Mean xenograft volume of 5 to 27 mice ± SEM are shown in mm^3^. p<0.05 (^*^); p<0.01 (^**^); p<0.001 (^***^); p<0.0001 (^****^).

To investigate the systemic effects generated by a primary cervical carcinoma xenograft on the growth of a secondary xenograft implanted at a distant anatomical site, we first developed an s.c. double xenograft model in athymic nude mice. This model consisted of two consecutively s.c. implanted xenografts by inoculation of the HPV-16 cervical carcinoma SiHa cell line at two different anatomical sites. The first xenograft [XNG (A)] was implanted into the lower right flank of the mice while the second one [XNG (B)] was induced 4 weeks later by injection of SiHa cells into the left dorsal flank (Figure 1A). The primary tumors were visible and measurable after 1 week post-inoculation of the tumor cells and reached a volume of about 600 mm^3^ within 4 weeks, the time point at which the second xenograft was induced (Figure 1B). The primary xenografts continued their exponential growth and did not affect the growth of the secondary xenografts induced at a distant anatomical site. Thus, the growth of XNG (B) was comparable among animals bearing both xenografts [i.e. XNG(A) / XNG(B)] and those in which only one xenograft was induced into the left dorsal flank [i.e. XNG(B)].

### Cidofovir *in situ* treatment of a primary xenograft resulted not only in a local antitumor effect but also in a temporary far-reaching (FR) effect on the growth of a secondary distant tumor

Chemical agents, radio-, cryo-, and photodynamic-therapy, high-temperature ablation (e.g. laser, ultrasound) and electric-based ablation are used for tumor ablation of localized malignancies. *In situ* tumor treatment may release tumor-associated antigens and danger signals that activate the immune system [16, 32]. This enhances anti-tumor responses resulting in the destruction of residual malignant cells in primary tumors and distant metastases. Therefore, we focused on the activation of anti-tumor immunity following ablation of the SiHa xenografts by intratumoral (i.t.) treatment with cidofovir. The s.c. double xenograft model was used to evaluate whether local treatment with cidofovir of the primary tumor would induce a FR effect leading to a reduction in the growth rate of an untreated distant xenograft. Intratumoral cidofovir treatment (25 μl of a 10 mg/ml solution 5x/week) of the first SiHa cervical carcinoma xenograft was started one week post-inoculation of the tumor cells and lasted for a period of 3 weeks. Intratumoral administration of PBS or needle puncture served as placebo and mock-treatment, respectively. At the time CDV, PBS and needle puncture i.t. treatment ended, the animals were inoculated with SiHa cells at the left dorsal flank to induce the secondary xenograft which remained untreated. The growth of the primary xenograft was not affected by either mock- (needle puncture) or PBS- (placebo) treatment compared to untreated xenografts (Supplementary Figure 1A). Neither was the growth rate of the secondary tumor (Supplementary Figure 1B). In contrast, cidofovir established a significant reduction of the primary xenograft growth, which was already visible after 1 week of treatment (Figure 1C). This local antitumor effect of cidofovir reached its maximum from week 3 to 6 and was still present at week 8, indicating a long-lasting anti-tumor action as cidofovir-treated tumors had a size smaller than 250 mm^3^ even when treatment was stopped. Notably, the most important differences in XNG (A) volume between PBS- and cidofovir-treated mice (i.e. 8.9 fold) were observed at week 6 (two weeks after *in situ* tumor treatment was halted). At this time point, cidofovir treatment of the primary xenograft induced a FR effect that resulted in a decreased growth rate of a secondary untreated xenograft implanted at a distant location (Figure 1D). XNG (A)-CDV treated mice had secondary xenografts that grew 2- to 2.5-fold slower than those of XNG (A)-PBS treated animals or mice that were only implanted with a xenograft into the left dorsal flank [i.e. XNG (B) group], respectively.

In summary, these results indicated that *in situ* tumor treatment with cidofovir was not only capable of significantly diminishing the volume of the treated primary tumor but also of inducing a FR effect that reduced the growth rate of a secondary distant xenograft. The (FR) effects of cidofovir should be considered to be the consequence of an indirect mechanism and not linked to a systemic release of the drug following intratumoral administration. This is sustained by our previous results showing that systemic treatment with high doses of cidofovir did not result in reduction of SiHa cervical carcinoma xenograft growth in nude mice [28, 33]. To gain more insights into cidofovir FR effects, several parameters linked to the pathology provoked by the growth of cervical cancer xenografts in the double xenograft mouse model were examined.

### Cidofovir *in situ* treatment of a primary xenograft diminished the pathology associated with total tumor burden

We first analyzed the mortality associated with the total tumor burden, including also animals that had to be euthanized because of ethical reasons [i.e. mice bearing a total tumor volume ≥ 2000 mm^3^]. In mice with only a xenograft induced s.c. in the left dorsal flank at week 4 and in those with double s.c. xenografts with the primary tumor being treated with cidofovir, no deceased mice were registered through the entire experiment (Figure 1B and 1C). In contrast, among animals that bore two s.c. xenografts with the first one remaining untreated or PBS-treated, 6 and 9 mice, respectively, died between weeks 5 and 8 (Figure 1B and 1C).

**Figure 2:**
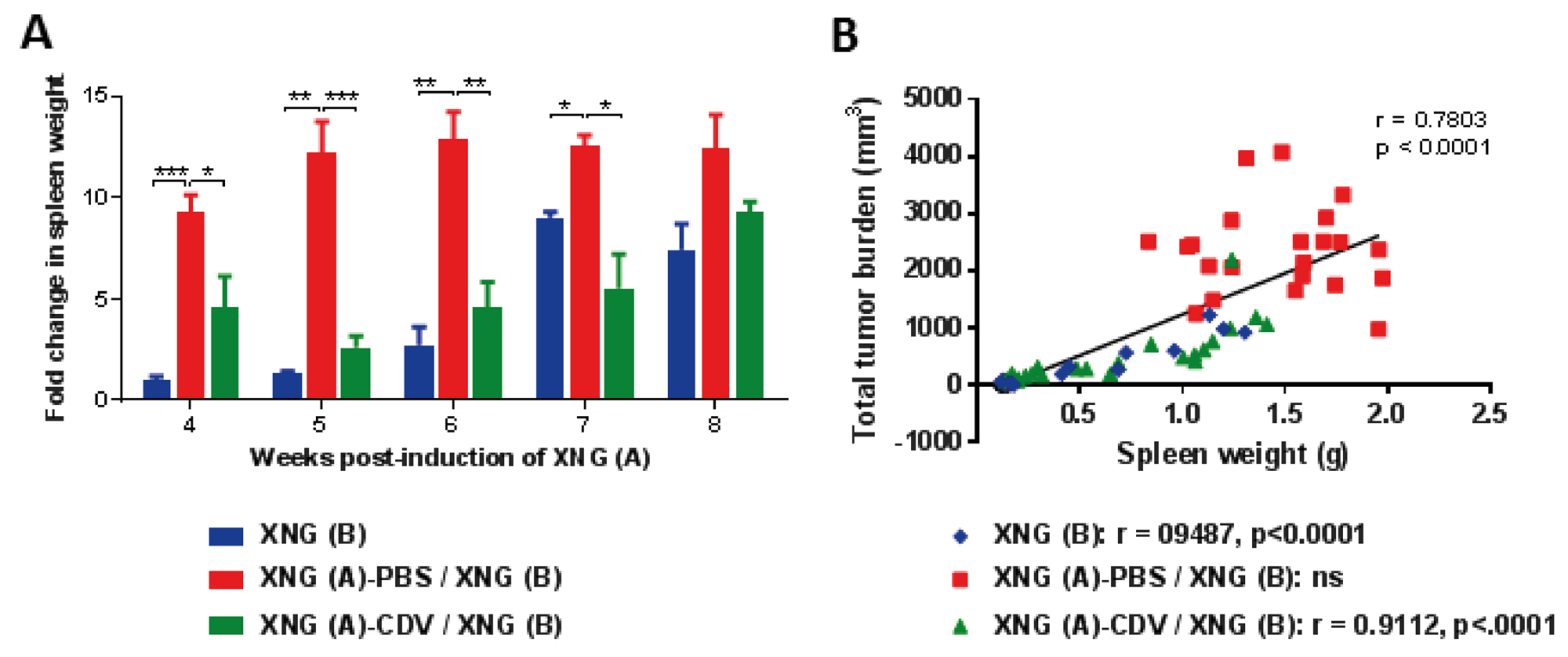
Development of splenomegaly **(A)** Fold change in spleen weight of mice that were induced SiHa cervical carcinoma xenograft(s) relative to control healthy mice, which had an average spleen weight of 0.1 g. Spleens were weighed immediately after dissection of the animals (N=3-5 mice per group). Statistical significance was indicated as follows: p<0.05 (^*^); p<0.01 (^**^); p<0.001 (^***^). **(B)** Pearson correlation of spleen weight (g) *versus* the sum of XNG (A) and (B) volume (mm^3^) for the three cohorts altogether. The Pearson correlation coefficient (r) and p-value for each individual group is indicated in the lower part of the figure.

Because we previously reported the development of splenomegaly in nude mice bearing a single SiHa cervical carcinoma xenograft and a reduction of splenomegaly following cidofovir intratumoral treatment [28], spleen weight was also recorded in the experiments described here. A highly significant splenomegaly was recorded at all evaluated time points (weeks 4 to 8) in the untreated group [XNG (A) / XNG (B)] and the placebo group [XNG (A)-PBS / XNG (B)] compared to control animals (no xenografts induced) (data not shown and Figure 2A). In the placebo group, the increase in spleen weight relative to healthy control mice ranged from 9- to 12-folds over time. Mice that received local treatment of the primary xenograft with cidofovir [i.e. XNG (A)-CDV / XNG (B)] had a substantial reduction in splenomegaly compared to the placebo group at each analyzed week, except for week 8 when the growth of the secondary xenograft became prominent. Notably, until week 6, splenomegaly in the cidofovir group remained stable (2.7- 4.0 fold enlargement of the spleen compared to control animals). This again indicated a transient FR effect of cidofovir treatment of a primary xenograft on the pathology induced by the growth of both the first and second implanted xenografts. The highest difference in splenomegaly between the placebo and cidofovir groups was seen at week 5, with 11.1-fold (placebo) *versus* 2.7-fold (cidofovir) increase in spleen weight. Mice that only bore a xenograft implanted at week 4 [i.e. XNG (B)] only started to develop splenomegaly from week 6 onwards and had significantly smaller spleens compared to the XNG (A)-PBS / XNG (B) group from weeks 4 to 7 but not at week 8 (once the tumor size of XNG (B) reached a considerable volume). A significant correlation between total tumor burden and spleen weight was found when the three groups were analyzed jointly (Figure 2B). However, when considered individually, the total tumor burden was correlated to development of splenomegaly in the cidofovir and XNG (B) groups but not in the placebo or untreated cohorts (Supplementary Figure 2). This indicated that in the latter groups the animals had already reached a severe spleen pathology at week 4 post-induction of the first xenograft. In contrast, in naïve animals [i.e. XNG (B) group that had implanted the tumor cells at week 4] as well as in cidofovir-treated mice, no or low spleen pathology, respectively, was evidenced at week 4, and from that time on, splenomegaly developed concomitant with tumor growth.

These data signified that cidofovir local treatment of a primary xenograft led not only to a temporary reduction in the growth of a second induced xenograft but also to a decrease in the pathology associated with tumor(s) growth, highlighting a (FR) anti-tumor effect of the drug.

### Reduction in enhanced recruitment of neutrophils to diverse organs following cidofovir intratumoral treatment of a primary xenograft

**Figure 3:**
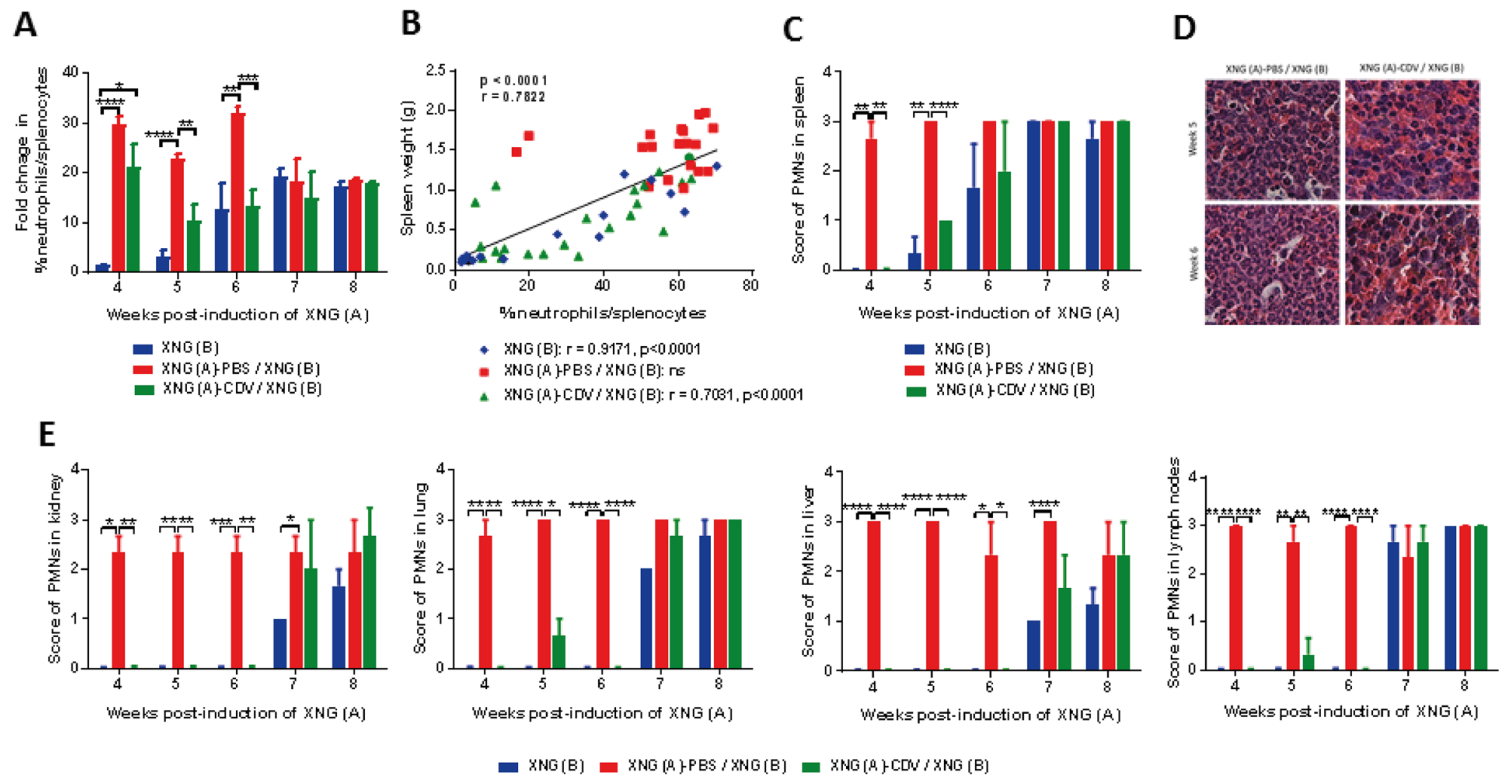
Polymorfonuclear (PMN) cell infiltration in spleen, kidney, lung, liver and lymph nodes **(A)** Fold change in the percentage of splenic neutrophils compared to control healthy mice, which had an average neutrophil percentage of 2 to 10 % in their spleen at weeks 4 to 8 (N=3-5). Percentages of neutrophils were obtained by performing flow cytometry on single cell suspensions from the spleen. A fixed order of gating was used: first a gate was drawn to include the correct cell population and to exclude debris, a second gate excluded doublets. Next, a gate was set on the living cells, followed by a gate on the CD45^+^ population (leukocytes). Neutrophils were identified as CD45^+^/GR1^+^/CD11b^+^ cells. **(B)** Pearson correlation between spleen weight (g) and the percentage of neutrophils in spleen for the three cohorts altogether. The Pearson correlation coefficient (r) and p-value for each individual group is indicated in the lower part of the figure. **(C)** Score of PMN infiltration in spleen (N=3) and **(D)** representative picture (40X magnification) of PMNs in spleen of mice from groups XNG (A)-PBS / XNG (B) and XNG (A)-CDV / XNG (B) at weeks 5 and 6. **(E)** Score of PMN infiltration in kidney, lung, liver and lymph nodes. The percentage of PMNs in tissue slides was microscopically (10X magnification) estimated and semi-quantified using the following scale: 0 score for ≤ 5% PMN infiltration, 1 for 6-25%; 2 for 26-49%; and 3 for ≥ 50%. The PMN score in all organs of control mice was 0. Values are shown as mean ± SEM (N=3). p<0.05 (^*^); p<0.01 (^**^); p<0.001 (^***^); p<0.0001 (^****^).

To explain the FR antitumor effects of cidofovir, we first examined immune cell activation in various organs. Neutrophils, one of the first cell types recruited to the sites of infection [34], have an important role in multiple aspects of cancer biology [35, 36]. To investigate whether cidofovir would affect neutrophil infiltration in our subcutaneous double xenograft model, a double staining with Gr1- and CD11b-specific mAbs followed by a flow cytometry analysis was carried out. Single cell suspensions prepared from spleens resected at diverse time points post-induction of the first xenograft were analyzed. We recorded a marked accumulation of Gr1^+^/CD11b^+^ cells over the total number of splenocytes in the XNG (A)-PBS / XNG (B) group compared to control healthy animals (Figure 3A). The percentage of neutrophils was in the range of 2-4% of the total splenocytes in healthy animals and raised to 23-68% in the XNG (A)-PBS / XNG (B) group throughout weeks 4 to 8. In the cidofovir group, this enhanced recruitment of neutrophils to the spleen was significantly reduced between weeks 5 and 6 (i.e. 2 weeks after compound administration was halted), with 10 to 13-fold neutrophil accumulation in the cidofovir group *versus* 23 to 32-fold in the placebo group (Figure 3A). At weeks 7 and 8, no differences in spleen neutrophil infiltration between both groups were measured. Thus, the effects of cidofovir on the reduction of spleen neutrophil infiltration coincided with its temporary FR antitumor effects leading to a delay in the growth of the secondary xenograft. Spleen neutrophil accumulation was parallel with splenomegaly as a strong correlation between spleen weight and the percentage of neutrophils was observed when the different cohorts were analyzed altogether (Figure 3B). Similar to the spleen weight *versus* total tumor burden association, a strong correlation between neutrophil increase and splenomegaly was calculated for the XNG (B) and cidofovir cohorts but not for untreated or PBS-treated mice (Supplementary Figure 3).

Our flow cytometry data on splenic neutrophil infiltration were confirmed by histopathological analysis of hematoxylin/eosin (HE) stained spleens (Figure 3C and 3D). We could observe polymorphonuclear cells (PMNs) infiltrating the spleens and we used a semi-quantitative method and a numerical scale with score 0 for ≤ 5% PMN infiltration, 1 for 6-25%; 2 for 26-49%; and 3 for ≥ 50%. There was a good correspondence between the flow cytometry and histology data, regarding the total PMN infiltration at all weeks except for week 6, in which no significant differences between groups were recorded by histological analysis while they were detected by flow cytometry.

Tumor growth not only promoted a strong accumulation of PMNs in the spleen but also in other organs (i.e. kidney, lung, liver, and lymph nodes), which was strongly impaired following local treatment of the primary xenograft with cidofovir (Figure 3E). This cidofovir effect was sustained until week 6 (i.e. 2 weeks after cidofovir therapy was halted) and coincided with the delay in growth rate of the secondary xenograft. Indeed, the pattern of accumulation of PMNs in different organs was comparable between the cidofovir cohort and the group bearing only a XNG (B). These data also suggested that cidofovir treatment of the first xenograft resulted in an overall reduced inflammation in the animals because of a decreased tumor burden compared to the placebo group.

### The reduction in splenic macrophages, B cells and NK cells associated with SiHa xenograft growth was reverted following cidofovir *in situ* treatment of a primary xenograft

**Figure 4:**
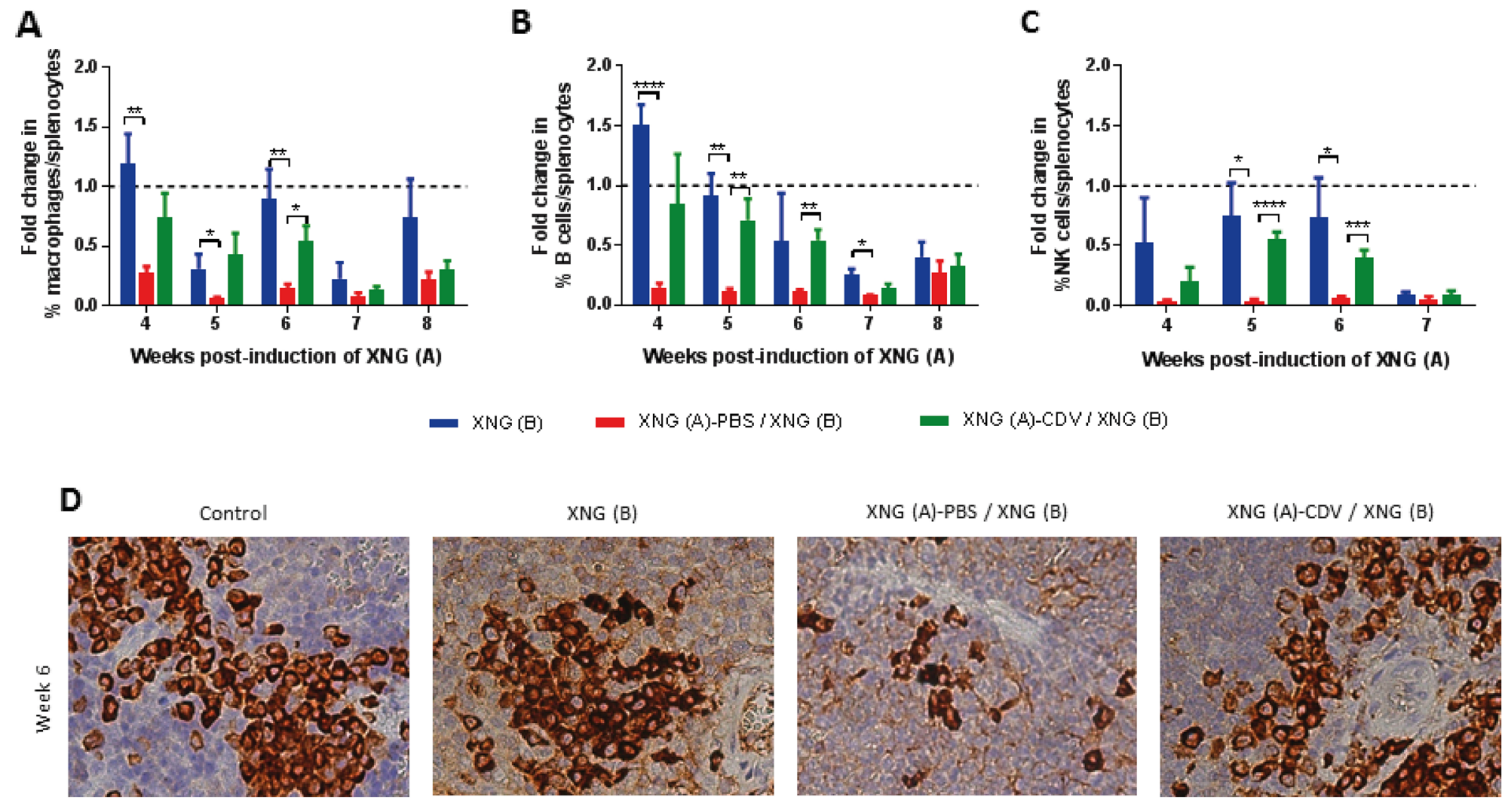
Infiltration of macrophages, B cells and NK cells in the spleen Fold change in the percentage of macrophages **(A)** B cells **(B)** and NK cells **(C)** compared to healthy control animals. The percentage of these immune cells was obtained by performing flow cytometry on single cell suspensions from the spleen. Macrophages were identified as CD45^+^/F4/80^+^/CD11b^+^ cells, B cells as CD45^+^/B220^+^/CD19^+^ cells NK cells as CD45^+^/CD49b^+^/CD3^-^ cells. Control mice had on average 0.7-2.7% macrophages, 26-37% B cells and 2-4% NK cells in their spleen. Values are shown as mean ± SEM (N=3-5). p<0.05 (^*^); p<0.01 (^**^); p<0.001 (^***^); p<0.0001 (^****^). **(D)** Representative picture (40X magnification) of macrophages in the spleen of mice in control healthy animals, mice with only XNG (B), mice with XNG (A)-PBS / XNG (B) and mice with XNG (A)-CDV / XNG (B) at week 6. Macrophages were detected by immunohistochemically staining using the primary antibody anti-F4/80.

Since macrophages are a highly heterogeneous population, with multiple functions and diverse phenotypes [37, 38], we explored the impact of local cidofovir treatment of a primary xenograft on this immune cell population in the spleen. Splenic macrophages were identified by expression of the pan macrophage marker F4/80, which is associated with macrophage differentiation, together with expression of CD11b, a marker of macrophage activation [39]. The population of F4/80^+^/ CD11b^+^ cells in the spleen was markedly reduced in mice bearing two xenografts relative to healthy control animals, in which the macrophages represented approximately 1 to 3% of the total splenocytes (Figure 4A). Cidofovir treatment of the primary xenograft was able to revert this profile, with significant differences between the placebo and cidofovir groups at week 6, when cidofovir FR effects were noticed. Immunohistochemistry for detection of F4/80^+^ cells confirmed that cidofovir was able to counterbalance the decline in splenic macrophage infiltration due to tumor growth (Figure 4D).

To differentiate between M1-like macrophages (effector cells for the elimination of pathogens, viral infected cells and malignant cells), and M2-like macrophages (cells that promote cancer cell proliferation, invasion, and metastasis by producing various mediators) [40, 41], we performed a staining to detect CD163, a marker for activation of tumor associated macrophages, i.e. TAMs or M2-like macrophages. F4/80^+^/CD11b^+^ /CD163^+^ cells were undetectable in healthy mice as well as in animals bearing SiHa cervical carcinoma xenograft(s), suggesting that TAMs do not play a role in our mouse model.

Although the B cell response is thought to be principally dependent on CD4^+^ T cell help, innate immune cells, including neutrophils, might promote the differentiation and activation of B cells independently of CD4^+^ T cells [42, 43]. To determine whether B cells could play a role in our nude mouse model lacking T cells, we analyzed the splenic B cell population by detecting CD19^+^/CD220^+^ cells. The percentage of B cells in the spleen of double tumor-bearing mice was markedly diminished compared to healthy animals (Figure 4B). Local treatment of the primary xenograft with cidofovir partially restored the splenic B cell levels. The highest differences in the splenic B cell population between the placebo and cidofovir groups were observed at weeks 5 and 6 post-inoculation of the first xenograft coincident with cidofovir FR effects.

The percentage of NK cells (CD49b^+^/CD3^-^) relative to the total number of splenocytes was also reduced in double xenograft-bearing mice compared to healthy animals (Figure 4C). Alike macrophage and B cell populations, *in situ* delivery of cidofovir to the primary tumor throughout weeks 1 to 3 post-inoculation of the tumor cells resulted in a reversion of the splenic NK cell population to physiological levels at weeks 5 and 6.

All these data pointed to a recovery of the physiological levels of macrophages, B cells and NK cells in the spleen following cidofovir *in situ* treatment of a primary xenograft in our s.c. double xenograft mouse model. The differences in the percentage of these immune cell populations between the placebo and cidofovir groups were significant at weeks 5 and 6, concomitant with the FR cidofovir effect. Furthermore, the splenic populations of macrophages, B cells and NK cells in mice that bore a single xenograft [i.e. XNG(B)] followed, for the most part, the patterns observed for the XNG (A)-CDV / XNG (B) animals (Figure 4). This implicated that i.t. cidofovir administration into a primary xenograft resulted in a decreased pathology associated with the total tumor burden in the double xenograft mouse model.

### Intratumoral administration of cidofovir to a primary xenograft led to a significant reduced recruitment of neutrophils and increased infiltration of macrophages, B cells and NK cells in the primary tumor, which were minor or absent in the secondary xenograft

**Figure 5:**
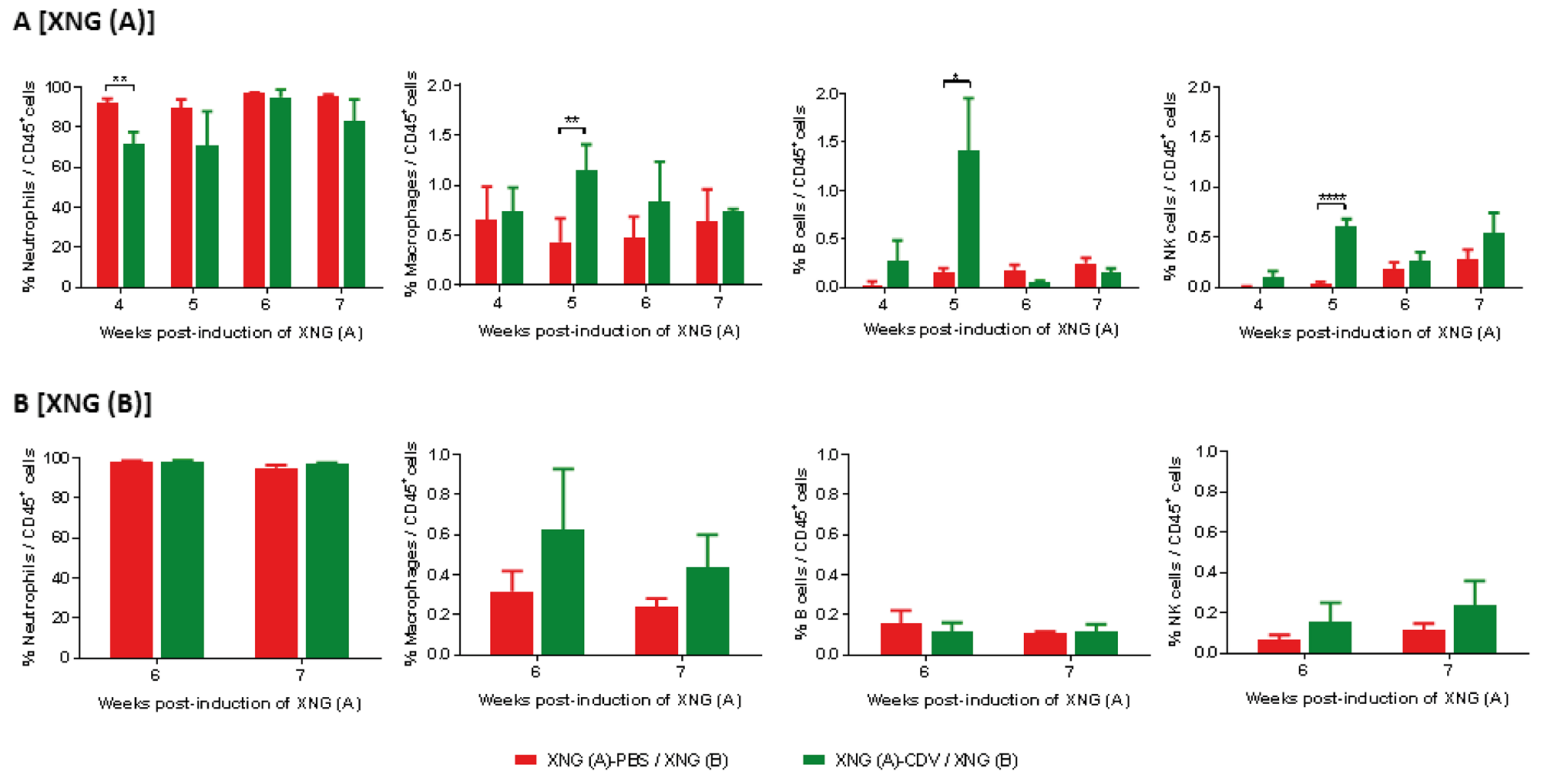
Infiltration of immune cells in primary **[XNG (A)]** and secondary **[XNG (B)]** subcutaneous SiHa cells xenografts. The percentage of neutrophils, macrophages, B cells and NK cells in **(A)** XNG (A) and **(B)** XNG (B) were obtained by performing flow cytometry on single cell suspensions from the xenografts. Neutrophils were identified as CD45^+^/GR1^+^/CD11b^+^ cells, macrophages as CD45^+^/F4/80^+^/CD11b^+^ cells, B cells as CD45^+^/B220^+^/CD19^+^ cells NK cells as CD45^+^/CD49b^+^/CD3^-^ cells. Values are shown as mean ± SEM (N=3-5). p<0.05 (^*^); p<0.01 (^**^); p<0.0001 (^****^).

Tumor-infiltrating immune cells in the xenografts were assessed using single cell suspension from tumors resected at different time points (Figure 5). High levels of intratumoral neutrophils have been significantly associated with unfavorable survival and recurrence in some human cancers [44]. In our model, the majority of the tumor-infiltrating immune cells proved to be neutrophils, with >90% of the CD45^+^ cells being neutrophils both in the primary and secondary xenografts. Cidofovir *in situ* treatment of XNG (A) led to a significant decrease in the percentage of neutrophils at week 4 in the primary tumor and the same trend was observed at week 5 although the difference with the placebo group was not significant due to deviations recorded among cidofovir-treated animals. In contrast, cidofovir had no impact on neutrophil infiltration in the secondary tumor. Differences in infiltration of macrophages, B cells and NK cells in the primary xenograft were observed at week 5 (i.e. one week after cidofovir treatment was discontinued), with their levels being significantly higher in the XNG (A)-CDV / XNG (B) cohort than in the XNG (A)-PBS / XNG (B) one. Although not statistically different, higher macrophage and NK cell infiltration in the secondary xenograft was detected at weeks 6 and 7 in the cidofovir group relative to the placebo.

### The decreased cytotoxic response of NK cells in mice bearing two xenografts was partially restored by cidofovir treatment

**Figure 6:**
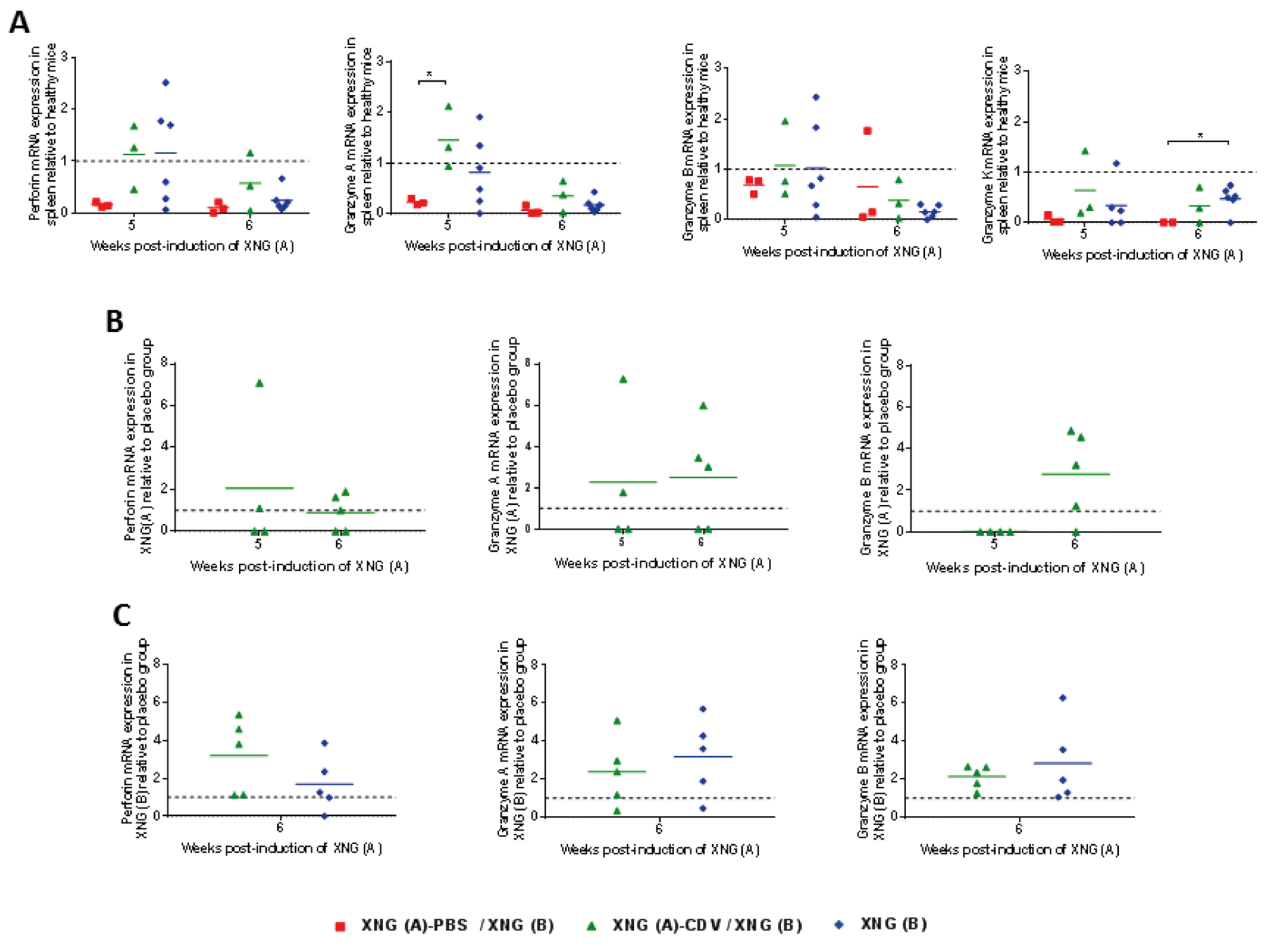
Levels of perforin and granzymes mRNA expression in spleen and s.c. xenografts Relative quantification of perforin and granzymes A, B and K mRNA expression in spleen **(A)**, XNG (A) **(B)** and XNG (B) **(C)** (N = 2-5) relative to control healthy mice (A) or placebo (B) and (C). GAPDH was used as housekeeping gene and all analyses were performed using GAPDH normalization. Relative gene expression was calculated using the 2^-ΔΔCt^ method. p<0.05 (^*^).

To investigate the effector cytotoxic response of NK cells, gene expression of the pore-forming protein perforin and of the serine protease granzymes were measured in spleen and xenograft extracts by quantitative RT-PCR (Figure 6). At week 5, the cidofovir group had an expression of granzymes and perforin that tended to attain those found in healthy animals, with statistical differences between the placebo and cidofovir groups seen for granzyme A gene expression. These data correlated with a normalization of the splenic NK cell infiltration observed in the XNG (A)-CDV / XNG (B) group (Figure 4C). Mice that only had a single xenograft induced at week 4 followed a similar pattern of granzymes and perforin expression as those found in the cidofovir cohort.

In the primary xenograft, about half of the animals in the cidofovir cohort had higher expression of perforin and granzyme A (week 5) and of perforin and granzymes A and B (week 6) than the placebo one, while granzyme K was undetectable in both groups (Figure 6B). This could be linked to a higher percentage of NK cells in the primary xenograft of cidofovir-treated mice than in the placebo-treated ones (Figure 5A).

When the second xenografts were analyzed, levels of perforin and of granzymes A and B were 1-to 7-fold higher in the cidofovir group than in the placebo group for most of the animals, and followed a pattern comparable to that of the XNG (B) cohort having a single xenograft implanted at week 4 (Figure 6C).

Altogether, these data pointed to a decreased NK cell infiltration and diminished effector cytotoxic response of NK cells in the spleen and xenografts of the placebo animals. The altered NK cell infiltration and cytotoxic response were partially counterbalanced following local cidofovir treatment of the primary xenograft, which could contribute to the reduced growth of a secondary untreated xenograft in the cidofovir cohort.

### Cidofovir FR effects were associated with diminished release of human IL-6 but not of IL-8 by SiHa cells

Most of the host- and tumor-derived cytokines and chemokines, evaluated in serum using a multiplex assay, were below the limit of detection or were detected at very low levels (i.e. ≤ 20 pg/ml). Among the mouse chemokines evaluated, only the TGF-1β chemokine was found at a serum concentration of 100-600 pg/ml but was not statistically different amongst the diverse groups (data not shown).

**Figure 7:**
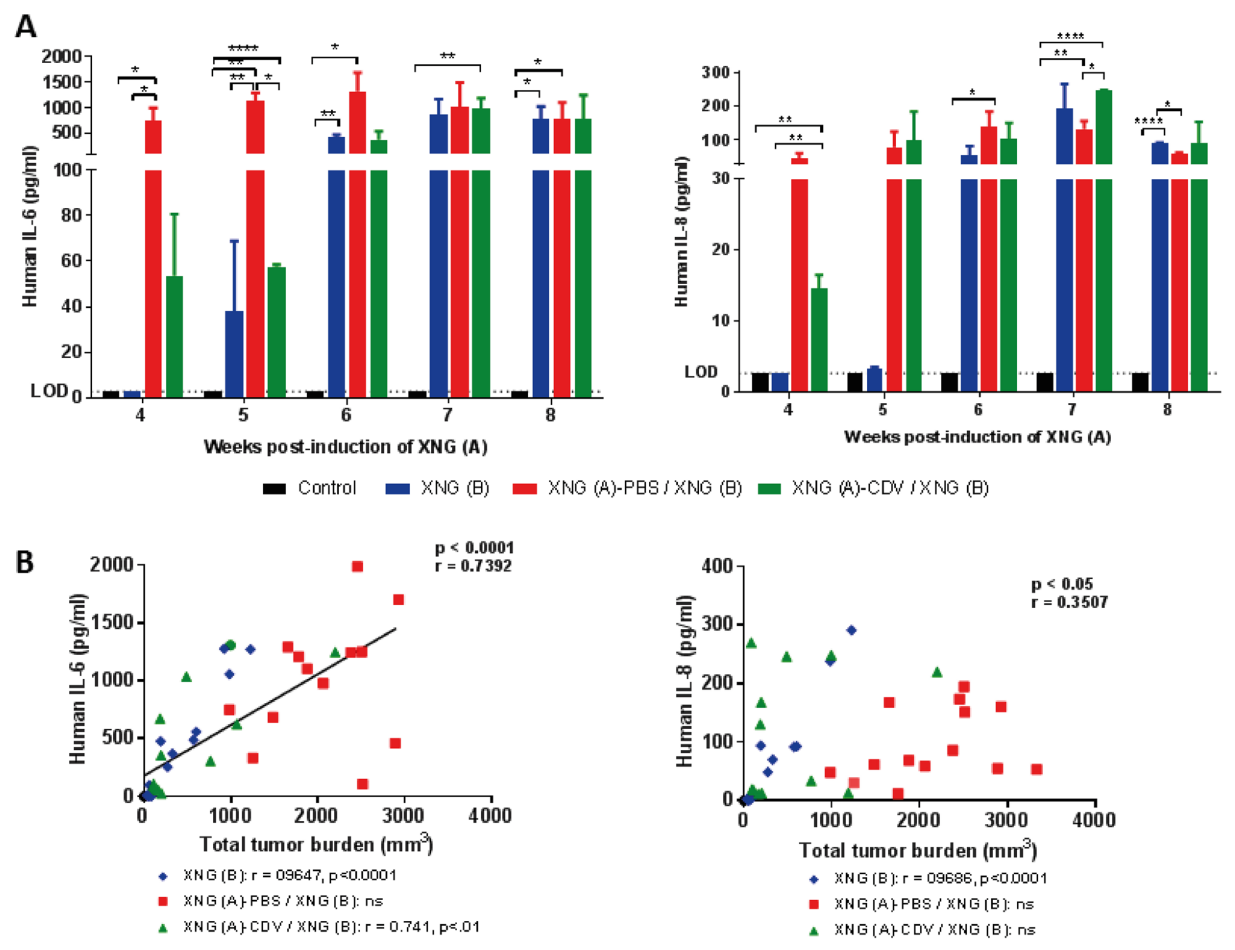
Human IL-6 and IL-8 levels in sera of mice **(A)** Levels of human cytokines (IL-6 and IL-8) determined by means of a Procartaplex assay. Data represent mean ± SEM (N=3) and the limit of detection (LOD) is shown. p<0.05 (^*^); p<0.01 (^**^); p<0.0001 (^****^). **(B)** Pearson correlation between serum cytokine levels (pg/ml) and the total tumor burden (mm^3^) for the three cohorts altogether. The Pearson correlation coefficient (r) and p-value for each individual group is indicated in the lower part of the figure.

With respect to human cytokines, only the inflammatory cytokines IL-6 and IL-8 were substantially secreted by SiHa tumor cells in the blood of mice (Figure 7). Importantly, mice induced with two xenografts that received *in situ* delivery of cidofovir into the primary xenograft had 14-fold (weeks 4 and 5) and 3.5-fold (week 6) lower serum levels of IL-6 than the placebo-treated animals. The IL-6 production by the cidofovir cohort was analogous to that of animals that were only induced with a single xenograft, except for week 4 (i.e. the time point at which the SiHa tumor cells were injected into the left dorsal flank and evidently, without detection of human IL-6 in the XNG (B) group). When cidofovir FR action fainted (i.e. from week 7 onwards), IL-6 release increased similarly in the cidofovir and placebo groups. The production of IL-6 was correlated with total xenograft(s) volume (Figure 7B) for the XNG (B) and cidofovir groups but not for the placebo or untreated groups (Supplementary Figure 4A).

In contrast to IL-6 serum secretion, no substantial differences in human IL-8 production between the placebo and cidofovir groups were found, with the exception of week 7. The production of this cytokine was not related to total tumor burden except for mice that only had a XNG (B) (Figure 7 and Supplementary Figure 4B).

These data indicated a role of human IL-6 and IL-8, known to be active in mice, as drivers of the pathology associated with the early growth of SiHa cervical carcinoma xenografts as evidenced by their correlation with tumor burden in animals having a recently implanted xenograft, i.e. XNG (B) group. Mice that had already an important tumor burden at week 4, i.e. placebo and untreated groups, had a total tumor burden which did not correlate with human IL-6 and IL-8 secretion throughout weeks 4 to 7. At the time that cidofovir FR effects were demonstrated, i.e. week 6, animals had diminished levels of IL-6 production but not of IL-8 compared to the placebo, indicating that cidofovir treatment of the primary xenograft resulted in reduced IL-6 production and inflammatory response.

### *In vitro* pretreatment of SiHa cells with cidofovir prior to implantation of a primary xenograft did not result in reduced growth of a secondary subcutaneous xenograft and slightly modified splenic immune cell infiltration

**Figure 8:**
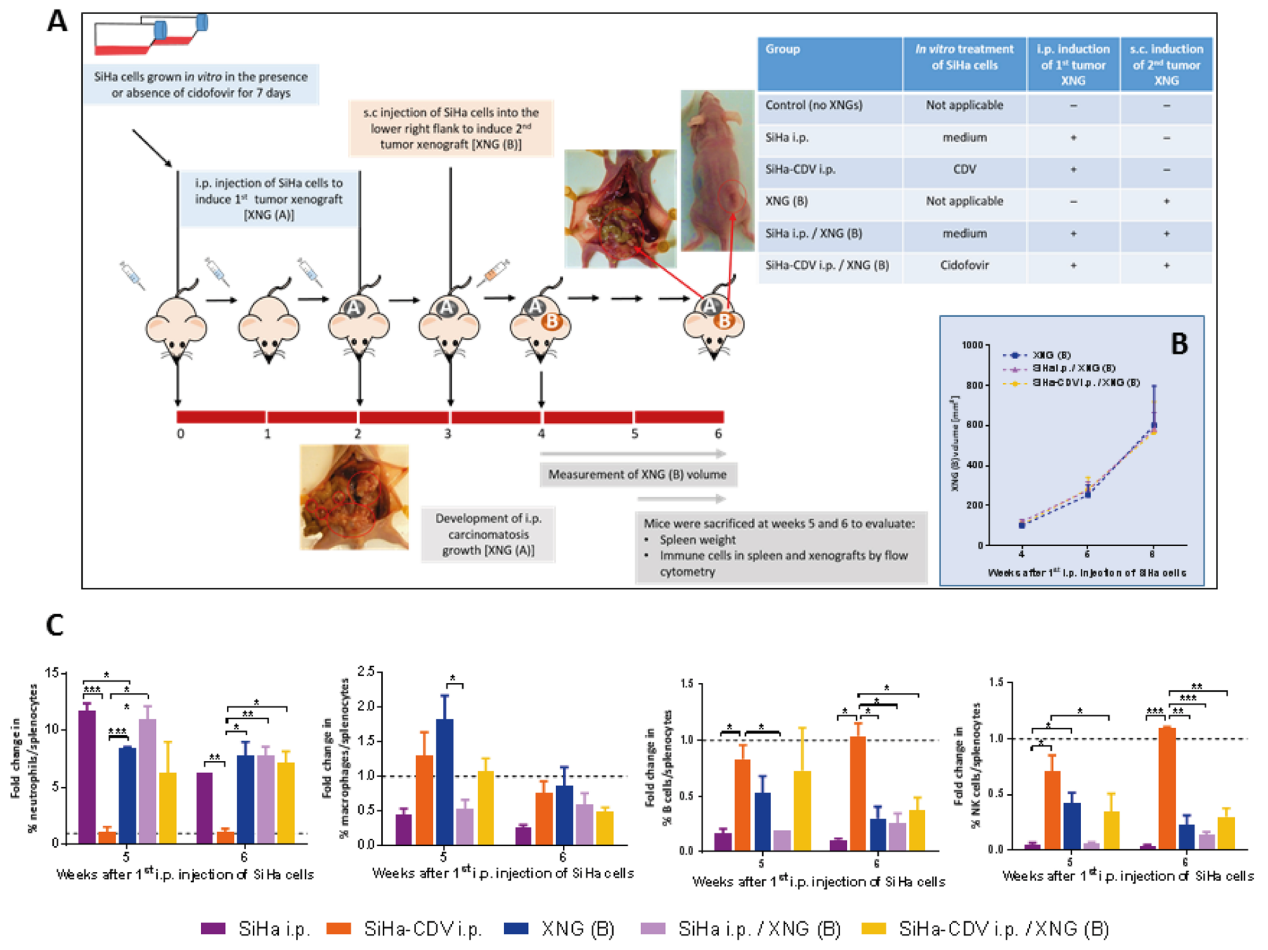
Tumor growth and splenic immune cell infiltration in the intraperitoneal / subcutaneous mouse model **(A)** In this model, the first tumor xenograft [XNG (A)] was induced by intraperitoneal (i.p.) injection of SiHa cells, which were *in vitro* pre-exposed or not to 50 μg/ml CDV for 7 days. SiHa cell suspension containing 4×10^5^ cells in 300 μl of PBS was injected i.p. to the mice twice per week for a period of two weeks. One week later, untreated SiHa cells were injected into the lower right flank of the mice to induce a secondary xenograft [XNG (B)]. Mice were euthanized at weeks 5 and 6 for evaluation of different disease parameters. **(B)** Xenograft B volume was measured once per week. Mean xenograft volume of 4 to 13 mice ± SEM is shown. **(C)** Fold change in immune cells compared to control healthy mice. Percentages of immune cells were obtained by performing flow cytometry on single cell suspensions from the spleen. Neutrophils were identified as CD45^+^/GR1^+^/CD11b^+^ cells, macrophages as CD45^+^/F4/80^+^/CD11b^+^ cells, B cells as CD45^+^/B220^+^/CD19^+^ cells, NK cells as CD45^+^/CD49b^+^/CD3^-^ cells. Values are shown as mean ± SEM (N=3). Control mice had on average 4-7% neutrophils, 1% macrophages, 28-35% B cells and 3-4% NK cells in their spleen. p<0.05 (^*^); p<0.01 (^**^); p<0.001 (^***^).

Apoptotic tumor cells are considered to be excellent sources for delivering a wide variety of antigens which induce an integral immune response [45]. To examine whether *in vitro* pretreatment of SiHa cells with cidofovir prior to implantation of a primary xenograft would expose antigens in the tumor cells which generate a systemic anti-tumor response, a second double xenograft model was developed (Figure 8A). In this model, a primary tumor was induced by intraperitoneal (i.p.) injection of SiHa cells (twice a week for a duration of 2 weeks) followed by s.c. challenge with SiHa cells 7 days after the last i.p. injection of tumor cells.

Firstly, we analyzed whether a different location of the primary tumor would affect the growth of a secondary tumor as location of a primary tumor was shown to have an impact on the prognosis of patients with metastatic disease (as shown in metastatic colorectal cancer patients with different Kras status receiving cetuximab) [46-48]. Following i.p. inoculation of SiHa cells, animals developed multiple peritoneal carcinomatosis which, similarly to s.c. inoculated SiHa cells, did not alter the growth rate of a secondary s.c. implanted xenograft (Figure 8B).

Next*, in vitro* pretreatment of SiHa cells with cidofovir, which is known to induce apoptosis in these cells [49], was assessed. Mice that were injected i.p. with *in vitro* cidofovir-exposed SiHa cells did not develop intraperitoneal carcinomatosis and therefore, splenic immune cell populations were similar to those of healthy mice (Figure 8C). This lack of development of intraperitoneal carcinomatosis can be explained by the relative high dose of cidofovir used to pretreat the cells (i.e. 50 μg/ml) and/or an easier elimination of the apoptotic tumor cells by the murine immune system. Nevertheless, cidofovir-pretreated SiHa cells were unable to delay the growth of a secondary xenograft implanted s.c. 2 weeks after induction of the primary xenograft (Figure 8B).

Development of SiHa cell carcinomatosis was associated with splenomegaly (Supplementary Figure 5), enhanced infiltration of splenic neutrophils and decreased macrophage, B cell and NK cell infiltration, which was absent in mice injected i.p. with cidofovir pretreated SiHa cells (Figure 8C). Mice that were injected i.p. with cidofovir-pretreated SiHa cells and that were induced a secondary s.c. tumor, displayed (at week 6) a level of splenic immune cell infiltration slightly lower (neutrophils) or higher (macrophages, B cells, NK cells) than the corresponding placebo group and similar to that of mice bearing a single XNG (B).

Immune cell infiltration of the secondary xenograft was comparable in animals bearing only a XNG (B) and in those that received i.p. untreated or cidofovir-pretreated SiHa cells (data not shown). These data were consistent with a lack of effect of cidofovir-pretreated SiHa cells on the growth rate of a secondary s.c. tumor and pointed to the inability of cidofovir-pretreated apoptotic tumor cells to expose antigens and generate a strong systemic anti-tumor response. However, at week 6, cidofovir-pretreated SiHa cells were able to revert marginally the splenic immune cell infiltration induced by the subcutaneous tumor (Figure 8C).

### The administration of cidofovir together with the adjuvants aluminum hydroxide and MPL did not boost cidofovir antitumor effects on the treated primary xenograft neither the FR effects on an untreated, distant secondary xenograft

**Figure 9:**
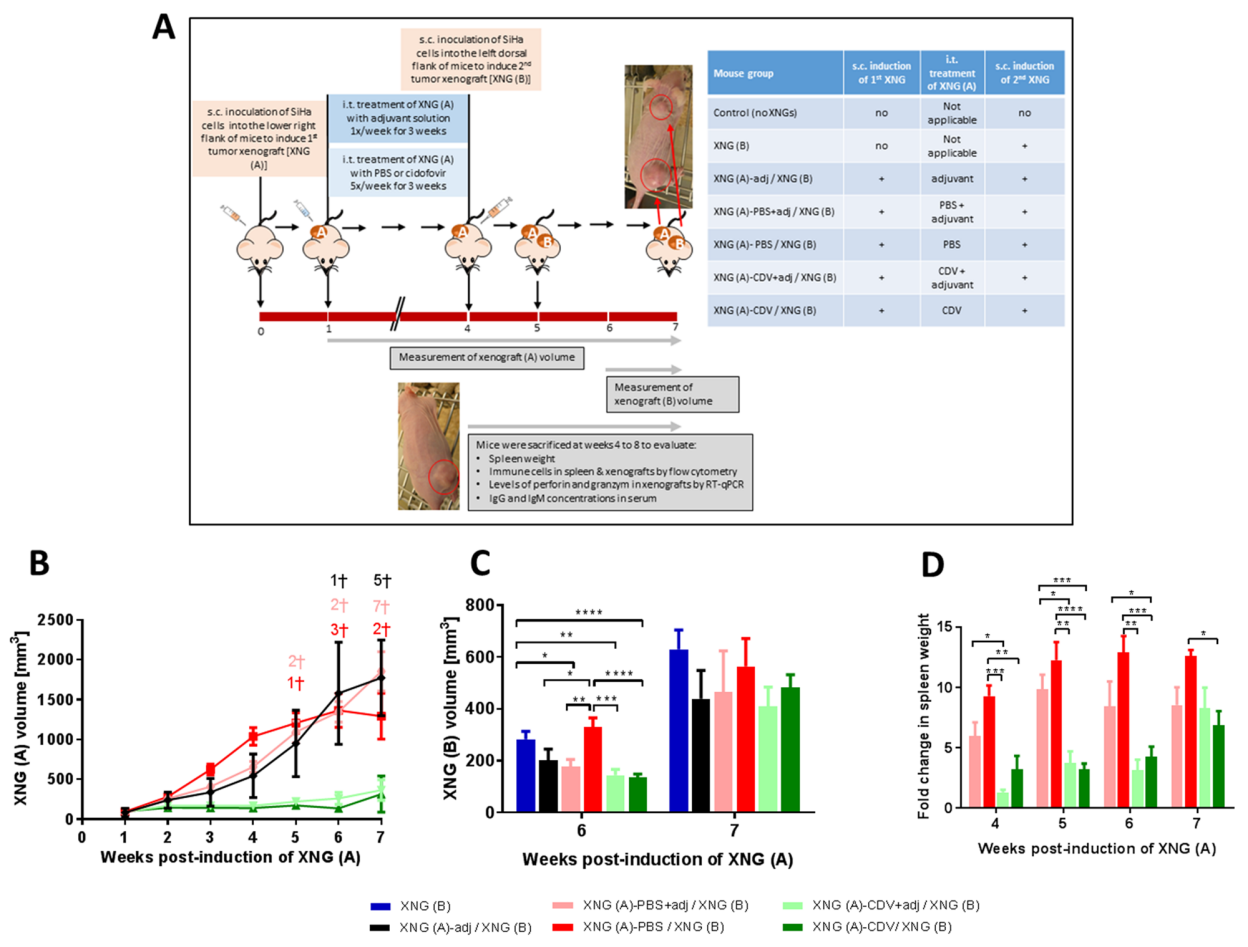
Effect of adjuvant treatment (aluminum hydroxide and MPL) on tumor growth in a double subcutaneous xenograft mouse model **(A)** Mice were inoculated s.c. into the lower right flank with 2×10^6^ SiHa cells in 200 μl PBS [primary xenograft, XNG (A)]. Intratumoral (i.t.) treatment with PBS or cidofovir started one week after injection of the SiHa cells and was performed 5 times per week for 3 weeks. Intratumoral adjuvant (adj) injections (0.5% alhydrogel and 0.05% MPL) were performed once a week for 3 weeks, simultaneously with PBS or CDV treatment. Four weeks after injection of XNG (A), a second xenograft [XNG (B)] was induced (2×10^6^ SiHa cells in 200 μl PBS) into the left dorsal flank. From week 4 to 7, mice were euthanized for evaluation of different disease parameters. Volume of XNG (A) **(B)** and XNG (B) **(C)**. Xenograft volume was measured once per week from week 1 [XNG (A)] or from week 6 [XNG (B)] onwards. Mice that died or had to be euthanized for ethical reasons are indicated on the graph by a cross. Mean xenograft volume of 6 to 27 mice ± SEM are shown in mm^3^. **(D)** Fold change in spleen weight of mice that were induced SiHa cervical carcinoma xenograft(s) relative to control healthy mice, which had an average spleen weight of 0.1 g. Spleens were weighed immediately after dissection of the animals (N=3 mice per group). p<0.05 (^*^); p<0.01 (^**^); p<0.001 (^***^); p<0.0001 (^****^).

A plethora of immunomodulatory substances has been evaluated for their capacity to enhance systemic antitumor immune responses [50]. In order to enhance cidofovir-induced tumor control, the drug was combined with the immune adjuvants that are used in the currently approved HPV vaccines, i.e. aluminum hydroxyphosphate sulfate (in Gardasil and Cervarix vaccines) and ASO4 (in Cervarix vaccine), a combination of aluminum hydroxide (Alum) and 3-O-desacyl-4’-monophosphoryl lipid A (MPL), a TLR4 ligand. We investigated the roles of the combination of MPL and Alum (MPL+Alum) in the s.c. double xenograft model. One week after induction of the first xenograft, animals received i.t. adjuvant injections [0.5% alhydrogel (an Alum wet gel suspension) and 0.05% MPL] once a week for a duration of 3 weeks (Figure 9A). An inhibitory effect of the adjuvant per se on the growth of the primary tumor was measured in week 3 and 4, with a 1.5 to 1.9 smaller xenograft volume in the groups that received only adjuvant or adjuvant and PBS *versus* PBS alone (Figure 9B). However, from week 5 onwards, treatment of the first xenograft with adjuvant had no impact on xenograft volume or mortality. Adjuvant treatment did not boost the anti-tumor effects of cidofovir on the primary xenograft as tumor size was comparable in the XNG (A)-CDV+adj / XNG (B) and XNG (A)-CDV / XNG (B) groups. In both groups, no deceased mice were registered and growth of the primary xenograft was significantly reduced from week 2 onwards compared to PBS-treated mice (regardless of adjuvant receipt).

The MPL+Alum combination per se also afforded a decreased growth of an untreated distant secondary tumor at week 6 (Figure 9C). However, the adjuvants did not boost cidofovir FR antitumor effects. Despite a transient effect of the adjuvants on tumor growth, splenomegaly was not significantly reduced when comparing adjuvant/placebo- *versus* placebo-treated mice (Figure 9D). In contrast, cidofovir afforded a significant reduction of the spleen size between weeks 4 and 6, irrespective of the presence of adjuvants.

**Figure 10:**
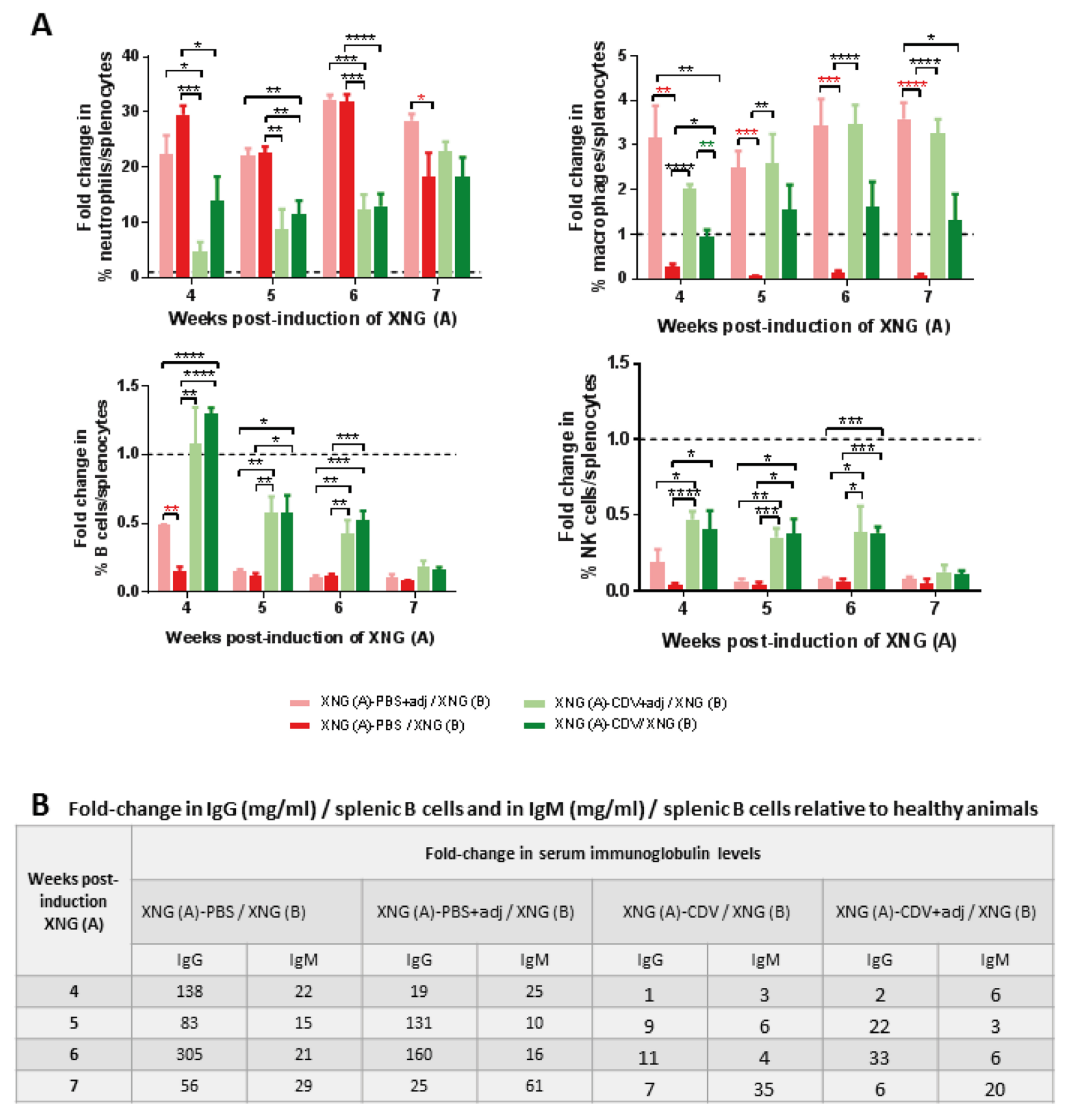
Effect of adjuvant treatment (aluminum hydroxide and MPL) on splenic immune cell infiltration and on serum immunoglobulin levels in mice bearing double SiHa cells xenografts **(A)** Fold change in immune cells compared to control healthy mice. Percentages of immune cells were obtained by performing flow cytometry on single cell suspensions from the spleen. Neutrophils were identified as CD45^+^/GR1^+^/CD11b^+^ cells, macrophages as CD45^+^/F4/80^+^/CD11b^+^ cells, B cells as CD45^+^/B220^+^/CD19^+^ cells, NK cells as CD45^+^/CD49b^+^/CD3^-^ cells. Values are shown as mean ± SEM (N=3). Control mice had on average 2-2.5% neutrophils, 2-2.7% macrophages, 28-37% B cells and 2-4% NK cells in their spleen. **(B)** Fold-change in ratio of serum IgG and IgM levels on splenic B cells compared to control healthy mice. Immunoglobulin levels were determined in the serum by means of ELISA. Results are shown as fold change of 3-5 values. p<0.05 (^*^); p<0.01 (^**^); p<0.001 (^***^); p<0.0001 (^****^).

The different splenic immune cells were evaluated, adjuvant treatment as such did not affect the neutrophil and NK cell populations but significantly enhanced the percentage of macrophages (at all tested time points) and of B cells (at week 4) compared to adjuvant-free placebo group (Figure 10). Adjuvant treatment did not alter cidofovir-induced effects on splenic B cells, NK cells and neutrophils, except for a slight decrease in the neutrophil/splenocyte ratio at week 4, though not statistically significant. The combination of adjuvant plus cidofovir also led to a boosted amount of splenic macrophages from week 4 onwards.

**Figure 11:**
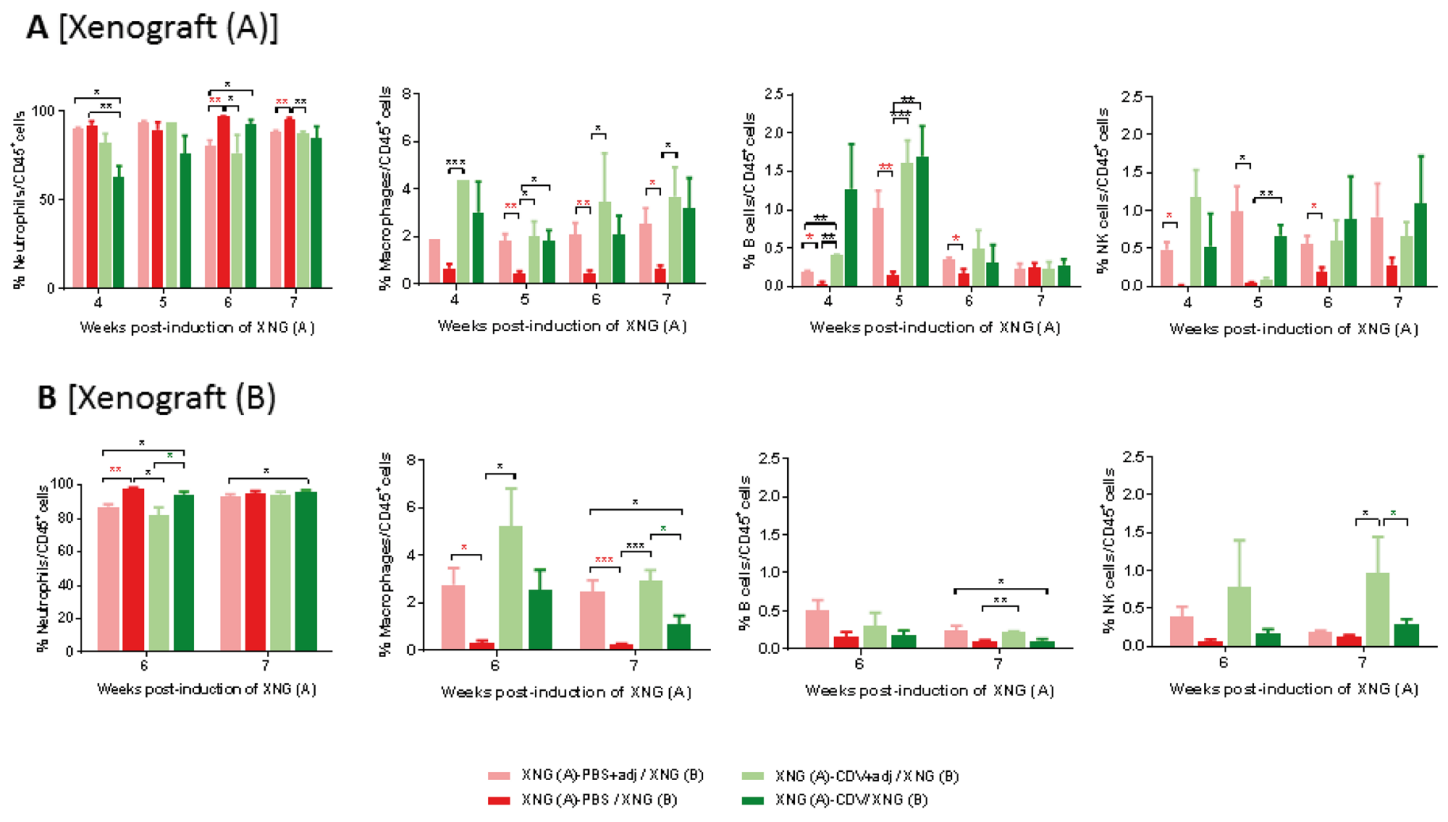
Effect of adjuvant treatment (aluminum hydroxide and MPL) on immune cell in primary [XNG (A)] and secondary [XNG (B)] subcutaneous SiHa cells xenografts The percentage of neutrophils, macrophages, B cells and NK cells in XNG (A) **(A)** and XNG (B) **(B)** were obtained by performing flow cytometry on single cell suspensions from the xenografts. Neutrophils were identified as CD45^+^/GR1^+^/CD11b^+^ cells, macrophages as CD45^+^/F4/80^+^/CD11b^+^ cells, B cells as CD45^+^/B220^+^/CD19^+^ cells NK cells as CD45^+^/CD49b^+^/CD3^-^ cells. Values are shown as mean ± SEM (N=3-5). p<0.05 (^*^); p<0.01 (^**^); p<0.001 (^***^).

Besides an effect on splenic immune cells, the MPL+Alum adjuvant treatment of the primary xenograft also affected (though not always statistically significant and sustained) the population of immune cells in the primary and/or secondary xenografts, with enhanced (NK cells, B cells and macrophages) or lessened (neutrophils) percentages compared to adjuvant-free groups (Figure 11). Consistent with a lack of adjuvant boost on cidofovir antitumor activity, the combination of adjuvant and cidofovir did not significantly modify the population of immune cells in cidofovir-treated primary xenografts (Figure 11A). However, the combination of cidofovir with MPL+Alum significantly reduced the neutrophil (week 6) and increased the macrophages and NK cell (week 7) populations in the secondary xenograft compared to the cidofovir group (Figure 11B).

When the cytotoxic response of NK cells was measured in the primary and secondary xenografts, the adjuvants per se did not markedly alter the expression of perforin and granzymes A and B (Supplementary Figure 6). Interestingly, cidofovir increased the NK cell cytotoxic response in the primary and secondary tumors regardless of the presence of adjuvants. Perforin and granzyme expression levels in XNG (B) were comparable between the cidofovir groups and the cohort having only a tumor on the left dorsal flank [i.e. XNG (B) group].

Considering that tumor burden was related to a reduction in the percentage of the splenic B cell population (CD45^+^/B220^+^/CD19^+^), which was counterbalanced by cidofovir treatment of the first xenograft, we evaluated whether mature B cells were activated and differentiated into antibody-secreting plasma cells. To estimate the activation and differentiation of B cells into plasma cells [CD138^+^, CD45 (B220)^low/-^, CD19^low/-^], we quantified the serum levels of IgM and IgG as T cell-independent B cell activation as secretion of IgM, IgG or IgA antibodies has been described in athymic nude mice [51]. In the double s.c. SiHa cells xenograft model, animals secreted considerable amounts of IgG and IgM compared to healthy controls, pointing towards an activation of the mature B cells to antibody-secreting plasma cells. We then calculated the ratios of IgM and IgG concentration to splenic B cells to estimate the proportion of activated B cells relative to mature B cells. Healthy animals had serum IgG concentration/splenic B cell ratios of 0.002-0.013 mg/ml, which were increased by 56- to 305-fold in the PBS group and by 19- to 160-fold in the PBS/adjuvant cohort. Importantly, when an effect of the adjuvants per se was detected on the growth of the primary tumor, i.e. week 4 (Figure 9B), the lowest fold-change of this ratio (i.e. 19-folds) for the XNG (A)-PBS+adj /XNG (B) group relative to heathy animals was found. The increases in the serum IgG concentration/splenic B cells ratios were of 1- to 11- fold in the cidofovir group and 2- to 33-fold in the cidofovir/adjuvant cohort), pointing to a reduced activation of B cells into plasma producing antibodies in cidofovir-treated mice (Figure 10B and Supplementary Figure 7). Similar results were found for IgM, though the increases in IgM serum concentration/splenic B cell ratios in the placebo groups (with and without adjuvants) relative to healthy animals were less pronounced than those found for IgG.

Overall, these data suggested that in this mouse model the activation of B cells is not associated with a clearance of the tumor cells. We may infer that following cidofovir treatment of the primary xenograft, a markedly diminished amount of tumor antigens are available resulting in a reduced activation of mature B cells into antibody-secreting plasma cells relative to placebo animals.

## DISCUSSION

Here we report that the growth of SiHa cervical carcinoma xenografts in athymic nude mice was associated with changes in the immune cell populations in the spleen that favored a tumor tolerant immune state. We showed that the presence of a primary cervical carcinoma xenograft had no impact on the growth of a secondary tumor xenograft induced at a distant anatomical site. However, ablative treatment of the primary xenograft by *in situ* delivery of the antiviral and antiproliferative drug cidofovir diminished the pathology associated with total tumor burden and resulted in a transient FR effect leading to decreased growth of an untreated distant secondary xenograft.

Because of the absence of T cells in athymic nude mice, these animals are commonly used for engraftment of human tumor cell lines with the advantage that their hairless phenotype allows easy assessment of s.c. tumor growth. We used tumor rechallenge experiments to test FR immune-mediated protection induced by *in situ* cidofovir delivery to a primary tumor. Our data point to the development of a cidofovir-induced protective systemic immunity since all rechallenged mice displayed long-term survival in contrast to untreated or placebo-treated mice. Tumor regression of the secondary xenograft was significant at week 6, i.e. 2 weeks after the end of cidofovir i.t. administration and 2 weeks post-tumor rechallenge. Immune-mediated tumor rejection has been shown to be dependent on several factors, including the amount of injected tumor cells, the time span between generation of antitumor immunity and rechallenge and anatomical location of the rechallenge [50, 52, 53]. A lower amount of cervical carcinoma cells used to induce the primary and secondary xenograft as well as different schedules of drug treatment and time of implantation of the secondary tumor could be further examined in the double s.c. SiHa cells xenograft model.

We may explain cidofovir FR effects by the induction of a number of systemic immune modulatory effects leading to decreased tumor growth. This phenomenon may arise from local cidofovir capacity to elicit these systemic immune effects to control the growth of a distant untreated tumor. We do not consider that a marginal release of the drug from the tumor in the blood may be responsible for the FR effect on a distant untreated tumor. We have previously demonstrated that i.p. administration of the drug five times per week with 25 μl of a 10 mg/ml cidofovir solution for 4 weeks was unable to diminish the growth of a cervical carcinoma xenograft [28, 33]. Moreover, cidofovir concentrations of 0.8 ± 0.2 μg/ml were determined in mouse sera 60 min after i.t. administration of 250 μg of the drug. Low cidofovir concentrations were also found in the blood of patients with recurrent respiratory papillomatosis after intralesional injections of cidofovir [54]. We hypothesize that cidofovir induces cell death in the *in situ* treated xenograft and the release of immunogenic factors, rendering the tumors accessible to immune cell infiltration. *In vitro* pretreatment of SiHa cells with 50 μg/ml cidofovir prior to i.p. administration to the animals failed to reduce the growth of a secondary s.c. xenograft. This concentration of cidofovir is known to induce apoptosis of SiHa cells [49]. Hence, we can speculate that after 7 days of *in vitro* exposure to the drug, the cells were (pre)apoptotic and unable to grow and to trigger an immune reaction, most likely because of a rapid elimination by the host immune system.

In the double s.c. tumor mouse model, neutrophils emerged as important contributors to the pathogenesis of SiHa cells xenografts. The FR effect of cidofovir was linked to a reduced number of neutrophils in the spleen and in the primary xenograft but not in the secondary xenograft when compared to the placebo cohort. Neutrophil function in cancer has been controversial as these cells were shown to possess a range of tumor promoting as well as tumor limiting properties [55, 56]. This debate may be explained by the fact that neutrophils are not a homogeneous population of cells and distinct neutrophil subsets may have different functions in the context of cancer as well as by the fact that neutrophils are highly responsive to changes in the microenvironment and may adopt a protumor or antitumor phenotype depending on the microenvironment [57]. A recent study identified intratumoral tumor-associated neutrophil density as an independent poor prognostic factor for survival in cervical cancer patients treated with radiotherapy [58]. Thus, an increased intratumoral density of tumor-associated neutrophils was significantly associated with shorter progression-free survival, lymph node metastasis, a lower complete response rate and a higher recurrence rate in these patients [58].

Because of the lack of T cells in our mouse model, only the impact of B cells and of innate antitumor responses could be evaluated. The restoration of the amount and activity of NK cells, primary cellular effectors of non-specific antitumor surveillance [59], in the cidofovir group was most pronounced at week 6, coincident with cidofovir FR effects. Several investigations have demonstrated that antitumor immunity can be enhanced by the augmentation of NK cell activity in different mouse models [60]. The activation of NK cells leads to the release of cytotoxic granules containing perforin and various granzymes and to cytokine production, mainly of interferon-γ [61]. The local antitumor and the FR effects of cidofovir were associated with a NK cell cytotoxic activity as demonstrated by the increased expression of perforin and granzymes in spleen as well as in primary and secondary xenografts.

A reduced growth of the primary xenograft by i.t. cidofovir resulted in less production of the human pro-inflammatory cytokine IL-6. It is well known that inflammation upregulates several immune protumor effector mechanisms, thereby preventing the immune system from rejecting malignant cells and providing a tumor-friendly environment that favors tumor growth [62]. Indeed, immune infiltration, activation of NK cells (as measured by expression of perforin and granzymes), activation of B cells into plasma cells (as determined by quantification of serum IgG and IgM levels) and levels of human IL-6 were comparable in the XNG (A)-CDV / XNG (B) group and the XNG (B) cohort (having been induced only a xenograft at week 4), and were significantly different from the placebo group.

In the double cervical cancer xenograft model, we can assume that inflammation induced extramedullary hematopoiesis. Under specific disease conditions, including cancer, splenic hematopoietic stem and progenitor cells (HSPCs) intensely expand producing progeny locally [63]. Studies of splenic hematopoiesis in several animal models of disease suggested that HSPCs, originally described in bone marrow, accumulate in high numbers in the splenic red pulp of diseased animals and are more distort toward myelopoiesis at the cost of erythropoiesis and lymphopoiesis [63]. Furthermore, the spleen is now positioned as an important extramedullary site able to continuously supply growing tumors with neutrophils and tumor-associated macrophages [64]. The contribution of the spleen to tumor growth has been highlighted in a mouse model of lung adenocarcinoma and it was sustained by data obtained from patients with invasive cancer [64]. Significantly higher numbers of splenic granulocyte/macrophage progenitors were found *ex vivo* in cancer patients compared with controls. Moreover, *in vitro* cultures of splenocytes of patients with cancer produced higher numbers of granulocyte/macrophage colonies than splenocytes of a control patient [64].

The combination of the adjuvants MPL and aluminum hydroxide with cidofovir did not result in a long-lasting cidofovir FR effect. However, the adjuvants as such had a transient inhibitory effect on SiHa xenograft growth that was associated, similar to cidofovir, with increased percentages of macrophages (throughout weeks 4 to 7) and B cells (only at week 4). However, adjunct treatment per se, unlike cidofovir, did not activate NK cells and triggered differentiation of B cells into antibody-secreting plasma cells. Aluminum hydroxide and MPL are mostly used as adjuvants in cancer vaccines though various lipids A have been used to treat animals with established tumors [65]. In animal models, the antitumor effect of LPS (lipopolysaccharide) and of the biologically active moiety, lipid A, was shown to be indirect and to rely on the induction of both an innate and specific immune response, leading to cytokine production [66, 67]. Lipid A derivatives affected tumor development by inducing necrosis as well as apoptosis of tumor cells. The efficacy of lipids A depended on the type of molecule and on the administration schedule but in general, increased survival was obtained, accompanied in some cases by tumor regression and cure [65].

In the present study, we have evaluated the contribution of immune cells to the FR effect of cidofovir. Yet, the influence of non-immune cells in the tumor microenvironment as well as of exosomes, extracellular vesicles involved in intercellular communication that are released by all cell types, including cancer cells [68], were not examined here but their roles deserve further investigation. The exosome content, including proteins, noncoding RNAs (microRNAs and long noncoding RNAs), messenger RNAs, DNA, and lipids, can mediate paracrine signaling in the tumor microenvironment. Importantly, exosomes can disseminate through the extracellular fluid to reach remote target cells, whose phenotypes can be influenced through the delivery of their content by regulating mRNA and protein expression [69]. The double s.c. tumor mouse model used here could be extremely valuable for the assessment of intercellular communication.

The cidofovir FR effects described in this study can be considered, at least in part, comparable to the abscopal effects of radiotherapy, which are believed to arise from the capacity of local radiotherapy to elicit systemic immune effects to control unirradiated tumor burden [50]. However, in the present study, we did not implant the two tumors simultaneously to evaluate true abscopal effects, but consecutively. Although radiotherapy kills cancer cells through direct and indirect effects of radiation, it occasionally induces an abscopal effect in which localized radiation treatment leads to elimination of metastatic cancer at a distance from the irradiated area due to the induction of an effective antitumor immune response. While high-dose radiation is associated with immune function suppression, low-dose radiation may have the opposite effect, stimulating immune system functions, which could account for the abscopal effects of radiotherapy [70]. This may explain why out of field effects following radiotherapy, which is generally used at high doses, are extremely rare. Approaches to combine innate immune stimuli with radiation have been proposed to enhance antitumor immunity [71, 72]. The combination of local radiotherapy and immune-modulation can boost local tumor control and cause distant (abscopal) antitumor effects through enhanced tumor-antigen release and antigen-presenting cell cross-presentation, improved dendritic-cell function, and enhanced T cell priming [72]. Importantly, it has been demonstrated that the combination of i.p. cidofovir with radiotherapy enhanced the radio sensitivity in Epstein-Barr virus (EBV)-related malignancies both *in vitro* and *in vivo* [73]. The combined treatment in nude mice led to a complete tumor remission without increasing toxicity in two human EBV-related cancer xenografts. Cidofovir also augmented radiation-induced DNA damage and, further, promoted glioblastoma cell death as demonstrated in two distinct intracranial xenograft models in mice [31]. Combination therapy of ionizing radiotherapy (the standard of care for glioblastoma in humans) with i.p. cidofovir significantly extended the survival of mice bearing intracranial glioblastoma tumors. A dramatic increase in phosphorylation of histone H2AX, a sensitive indicator of DNA double-strand breaks, was found when cidofovir was combined with ionizing radiation, thereby showing that the DNA-damaging effects of radiotherapy were exacerbated by cidofovir [31].

The current treatment for locally advanced cervical cancer is radiation in association with platinum salt-based chemotherapy. Local control rate with this treatment varies with cancer stage, from more than 95% in stage I to 60–85% for stage IV [74, 75]. However, about 30 to 40% of patients with similar prognostic factors do not respond similarly to comparable standard treatments [76]. Therefore, strategies to overcome chemoradio resistance in cervical cancer are needed and cidofovir adjuvant therapy may be of use. A phase I study showed encouraging results when combining cidofovir with standard radiochemotherapy in stage IB2-IVA cervical cancer patients where no major toxicity and interesting efficacy raised high hopes [77]. Our double subcutaneous mouse model could be of importance to evaluate and investigate radiotherapy in combination with cidofovir not only for cervical cancer but also for other HPV-related (such as head and neck squamous cell carcinoma) and HPV-unrelated human cancers. Nevertheless, one of the limitations of our model is the lack of T cells; hence, syngeneic mouse models able to evaluate the role of T cells should be developed.

Cidofovir displays antitumor activity not only against HPV-associated malignancies but also against non-viral induced cancers. Improved pharmacological formulations (including nanotechnology) of cidofovir could be foreseen to enhance the FR effects of the drug. In view of the accumulating evidence showing the immune system has a critical role in the process of tumorigenesis, an emerging concept for managing established cancers involves the identification of drug combinations that not only kill cancer cells but also influence the immune system to fight against cancer [78]. Cidofovir may be one of such drugs given that we proved here that it is not only able to decrease the volume of the locally treated xenograft but also to reduce inflammation and to direct the immune response in such a way to be favorable to the host.

## MATERIALS AND METHODS

### Cell culture

The human cervical carcinoma cell line SiHa, which contains an integrated human papillomavirus (HPV) type 16 genome (HPV-16, 1 to 2 copies per cell), was purchased from the American Type Culture Collection (LCG Standards, Molsheim Cedex, France) (ATCC, HTB35™). Cells were maintained in Dulbecco’s modified Eagle’s medium supplemented with 10% fetal calf serum (FCS), 1X non-essential amino acids, 1 mM sodium pyruvate, 0.3 mg/ml L-Glutamine, 10 mM HEPES, 100 U/ml penicillin and 100 μg/ml streptomycin (all from Gibco, Life Technologies, Merelbeke, Belgium) in a 5% CO_2_ humidified atmosphere at 37°C.

### Compounds

The acyclic nucleotide analogue cidofovir (CDV) or (*S*)-HPMPC, [(S)-1-[3-Hydroxy-2-(phosphonomethoxy)propyl]cytosine] was obtained from Gilead Sciences (Foster City, Ca, USA). The compound was dissolved in PBS at a concentration of 10 mg/ml. The adjuvant Monophosphoryl Lipid A from *S. minnesota* R595 (MPL-SM VacciGrade) (Invivogen, San Diego, USA) was dissolved in DMSO at a concentration of 2 mg/ml. Alhydrogel adjuvant 2%, an aluminium hydroxide wet gel suspension (Invivogen), was combined with MPL to a final concentration of 0.5% and 0.05%, respectively.

### *In vivo* studies

Female athymic nude mice (NMRI-nu) weighing 18 to 20 g and being 4 to 5 weeks of age (Janvier Breeding Center, Le Genest St. Isle, France) were housed in sterile cages under standard conditions (22°C, 50% relative humidity, 12-h light/dark cycles) and provided with food and water *ad libitum*. The studies were carried out according to national regulations and were approved by the Animal Experiment Ethical Committee of the KU Leuven (Permission number: P196/2013). Two mouse models were developed in order to investigate the interactions between distant tumors. In the first model, two SiHa cervical carcinoma xenografts were successively induced subcutaneously (s.c.) (Figure 1A) while the second model consisted of a first intraperitoneal (i.p.) induced tumor xenograft and a second s.c. induced xenograft (Figure 8A).

### Double subcutaneous xenograft model

Mice were injected subcutaneously (s.c.) with 2×10^6^ SiHa cells into the lower right flank (200 μl cell suspension in PBS per mouse) to induce the first tumor xenograft [XNG (A)]. The starting point of the experiment (week 0) was defined as the time when XNG (A) was induced (Figure 1A). One week after tumor cell inoculation, the tumor sizes were 30 to 100 mm^3^, and the mice were randomly assigned to 4 different groups. One group remained untreated, two groups received treatment of XNG (A) by intratumoral (i.t.) injection with either 25 μl of a 10 mg/ml CDV solution or 25 μl of PBS (placebo) while one group had the tumor punctured with a needle (mock-treatment). Animals received treatment 5 times a week for a period of 3 weeks. When i.t. treatment of XNG (A) was ended, a second tumor xenograft [XNG (B)] was induced by injecting 2×10^6^ SiHa cells in 200 μl PBS into the left dorsal flank of these 4 mouse groups. The same amount of cells was administered to a mouse group that had not been previously injected with SiHa cells. Another group of mice without being induced any xenograft was included as control. From week 4 until week 8, mice were euthanized every week with a lethal injection of Nembutal (Sodium Pentobarbital, Ceva, Belgium) to analyze different parameters associated with the growth of the tumor xenografts. In summary, 156 mice (from 3 independent experiments), subdivided into 6 groups, were analyzed (Figure 1A).

To evaluate the effects of aluminum hydroxide and MPL adjuvants in this model, two independent experiments were performed (Figure 9A). Intratumoral adjuvant (adj) injections (0.5% alhydrogel and 0.05% MPL) were performed once a week for 3 weeks, simultaneous with PBS or CDV treatment. A total of 126 mice, subdivided in 7 groups, were analyzed.

### Intraperitoneal - subcutaneous tumor xenograft model

Before implantation into the mice, SiHa cells were grown *in vitro* in the presence or absence of 50 μg/ml of CDV for 7 days (Figure 8A). A cell suspension containing 4×10^5^ cells [CDV-pretreated or untreated] in 300 μl PBS was injected i.p. to the mice (2 injections per week for 2 weeks). Week 0 was considered the week that the tumor cells were first injected i.p. into the mice. At week 3 (i.e. one week after the end of i.p. injection of the tumor cells), animals were challenged by s.c. injection of untreated SiHa cells (2×10^6^ cells in 200 μl PBS) into the lower right flank. Two and three weeks after induction of the second xenograft, animals were euthanized and spleen weight as well as the population of immune cells in spleen and xenografts were evaluated. For this model, 49 mice subdivided into 6 groups were included.

### Growth analysis of s.c. xenografts

Following s.c. inoculation of SiHa cells to induce a first and/or a second tumor xenograft, mice were monitored for tumor growth every week. Tumors were measured using a digital caliper in two directions (perpendicular diameters) and the formula V (volume) = (long diameter x short diameter^2)/2 was applied to calculate the tumor volume. Once the total tumor burden [sum of the volume of XNG (A) and XNG (B)] reached 2000 mm^3^, mice were euthanized for ethical reasons.

### Characterization of immune cells in spleen and tumor xenografts

At different time points, mouse spleens and xenografts were collected and disrupted in cold PBS containing 2 % FCS (washing buffer) and then passed through a 70 μm nylon cell strainer to obtain single cell suspensions. Red blood cells in the spleen were lysed by 5 min incubation at 37°C with NH_4_Cl solution (0.83% in 0.01 M Tris HCl, pH 7.2) and the remaining cells were washed with PBS 2%. Cells isolated from the xenografts and spleens were counted with a Bürker chamber and aliquots of 1×10^6^ cells were frozen at -80°C for RNA extraction. For flow cytometry analysis, 5×10^5^ cells were incubated with FcR blocking Reagent (Miltenyi Biotec, Bergische Gladbach, Germany). Cells were then washed with washing buffer and stained with the antibodies of interest. After a washing step, cells were fixed with 0.4% formaldehyde in PBS and the population of immune cells in spleen and xenografts was determined by flow cytometry analysis.

The following antibodies were used: anti-LY-6G(GR1)-FITC (11-5931), anti-CD19-FITC (11-0193), anti-CDV45R(B220)-PE (12-0452), anti-F4/80-PE (12-4801), anti CD-3-FITC (11-0031), anti-CD11b-APC (17-0112), and anti-pan-NK cells (CD49b)-APC (17-5971) from eBioscience, Vienna, Austria; anti-CD163-FITC (orb13303) from Biorbyt, Cambridge, United Kingdom; anti-CD45-PE (553081), anti-CD45-APC (561018), anti-CD45-PerCP-Cy5.5 (561869) from BD Biosciences, Erenbodegem, Belgium. Viability staining was performed with Live/Dead Fixable Aqua (Life Technologies).

All flow cytometry data were acquired on a LSRFortessa X20 flow cytometer (BD Biosciences) and analyzed with Flow Jo software version 10 (Flow Jo LLC, USA). To determine the population of immune cells, different gates were applied successively. A first gate was drawn to exclude cell debris, a second gate excluded doublets, and a third gate was set on living cells, followed by a gate on the CD45^+^ population (leukocytes). Neutrophils were identified as GR1^+^/CD11b^+^ cells and macrophages as F4/80^+^/CD11b^+^ cells. Cells that were F4/80^+^/CD11b^+^ and expressed the scavenger receptor CD163 were identified as tumor associated macrophages (TAM). Detection of NK cells was achieved by drawing a gate on CD49b^+^/CD3^-^ cells and B cells were identified as B220^+^/CD19^+^ cells.

### Determination of serum cytokine levels

Blood samples were obtained via cardiac puncture immediately after euthanasia. Blood was allowed to clot for 30 min at room temperature and serum was collected after centrifugation of the blood at 1,000 x g for 10 min. An additional centrifugation step at 10,000 x g for 10 min at 4°C was performed to remove lipids. Serum was aliquoted and stored at -80°C.

Both human and mouse cytokine levels in mice sera were measured using a multiplex assay (ProcartaPlex^®^ Multiplex Immunoassays, Affymetrix eBiosciences) according to the manufacturer’s protocols. For the mouse cytokines a 14-plex panel including G-CSF, GM-CSF, IFN-γ, IL-10, IL-15, IL17A, M-CSF, IL-1b, GRO-α, IL-6, MIP-1α, MIP-1β, RANTES and TNF-α was used. TGF-β1 was detected by ELISA with the Mouse LAP (latency-associated peptide) (TGF beta 1) Ready-SET-Go!™ Kit (Affymetric eBiosciences).

For detection of human cytokines, a 9-plex panel (IL-6, IL-8, GRO-α, IFN-γ, IL-10, IL-15, IL17A, TGF-α and TNF-α) was employed. Human TGF-β1 was detected with the Human LAP (TGF-beta1) Ready-SET-Go! ELISA kit (Affymetric eBiosciences).

### Reverse transcriptase quantitative PCR (RT-qPCR) analysis of perforin and granzymes from spleen and xenografts

RNA was extracted from 1×10^6^ splenocytes and xenografts using the PureLink^®^ RNA Mini Kit (Invitrogen, Life Technologies) according to the manufacturer’s protocol and aliquoted at -80°C. RT-qPCR was performed with qScript™ XLT One-Step RT-qPCR ToughMix^®^ (Quanta Biosciences, Gaithersburg, US) and the TaqMan primer/probe sets from Applied Biosystems, Life Technologies, for Perforin 1 (Mm00812512), granzyme A (Mm00439191), granzyme B (Mm00442834) and granzyme K (Mm00492530) using the 7500 Fast Real-Time PCR System (Life Technologies). GAPDH (TaqMan Rodent GAPDH control reagents, Applied Biosystems) was used as housekeeping gene and all analyses were performed using GAPDH normalization. Relative gene expression was calculated using the 2^-ΔΔCt^ method.

### Histopathology

Different organs (i.e. spleen, kidney, liver, lung and lymph nodes) from weekly euthanatized mice were fixed in neutral buffered formalin, subsequently embedded in paraffin and 5 μm sections were hematoxylin-eosin (H&E) stained and microscopically examined (AxioVision 4.8 Imaging System, Carl Zeiss, Oberkochen, Germany). The degree of polymorphonuclear (PMN) cell infiltration in each tissue section was scored based on 10X magnification images of spleen, liver, kidney, lungs and lymph nodes using the following scale: score 0 for ≤ 5% PMN infiltration, 1 for 6-25%; 2 for 26-49%; and 3 for ≥ 50%.

### Immunohistochemistry

Spleen paraffin sections were deparaffinized and dehydrated in a graded series of ethanol solutions. Samples were processed in the BOND Max autostainer using Bond™ Polymer Refine Detection system (Leica Biosystems, Diegem Belgium). The primary antibody anti-F4/80 (MCA497GA AbD Serotec, Kidlington, United Kingdom) for the detection of macrophages was used.

### Analysis of immunoglobulins in mice sera

Mouse immunoglobulins G (IgG) and M (IgM) were determined in serum of mice by ELISA using the Mouse IgG total Ready-SET-Go! Kit (Affymetrix, eBioscience) and the Mouse IgM total Ready-SET-Go! Kit (Affymetrix, eBioscience), respectively, according to the manufaturer’s protocol.

### Statistical analysis

Statistical analyses were performed with GraphPad Prism 6 software. Xenograft volume, number of immune cells, cytokine levels, immunoglobulins concentration, perforin and granzymes gene expression, and (immune)histological parameters were analyzed using the unpaired *t*-test. For correlation analysis, the Pearson’s correlation test was applied. Two variables were considered strongly correlated when the correlation coefficient (r) was ≥0.7 (positive correlation) or ≤-0.7 (negative correlation). A linear regression line was drawn only when a significant correlation was found. Statistical significance was indicated as: p<0.05 (^*^), p<0.01 (^**^), p<0.001 (^***^), p<0.0001 (^****^).

## SUPPLEMENTARY MATERIALS FIGURES


