# Supplementary material for: Investigation of tumor-tumor interactions in a double human cervical carcinoma xenograft model in nude mice
Source: Oncotarget. 2018 Apr 24;9(31):21978–2000. doi: 10.18632/oncotarget.25140 (PMC5955163; doi:10.18632/oncotarget.25140)
Supplement: Supplementary file 1 [file oncotarget-09-21978-s001.pdf]

Investigation of tumor-tumor interactions in a double human cervical carcinoma xenograft model in nude mice

SUPPLEMENTARY MATERIALS

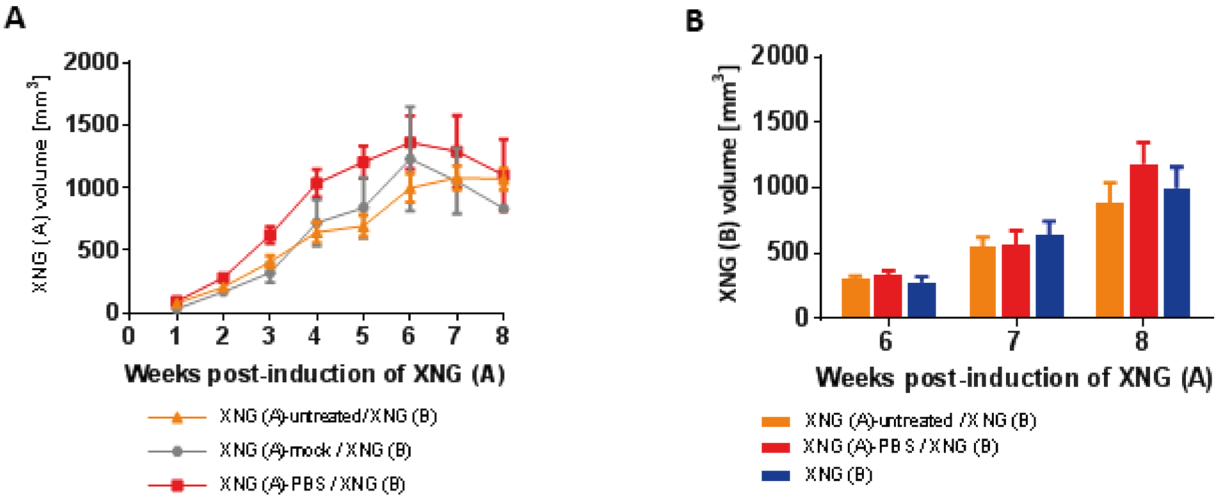

Supplementary Figure 1: Volume of primary **[XNG (A)]** and secondary **[XNG (B)]** tumors in a double subcutaneous xenograft mouse model. Growth of primary **(A)** and secondary **(B)** xenografts in mice of groups XNG (A)-untreated / XNG (B), XNG (A)-mock / XNG (B), XNG (B), and XNG (A)-PBS / XNG (B). Tumor volume was measured once per week. Mean xenograft volume of 3 to 27 mice ± SEM is shown in mm^3.

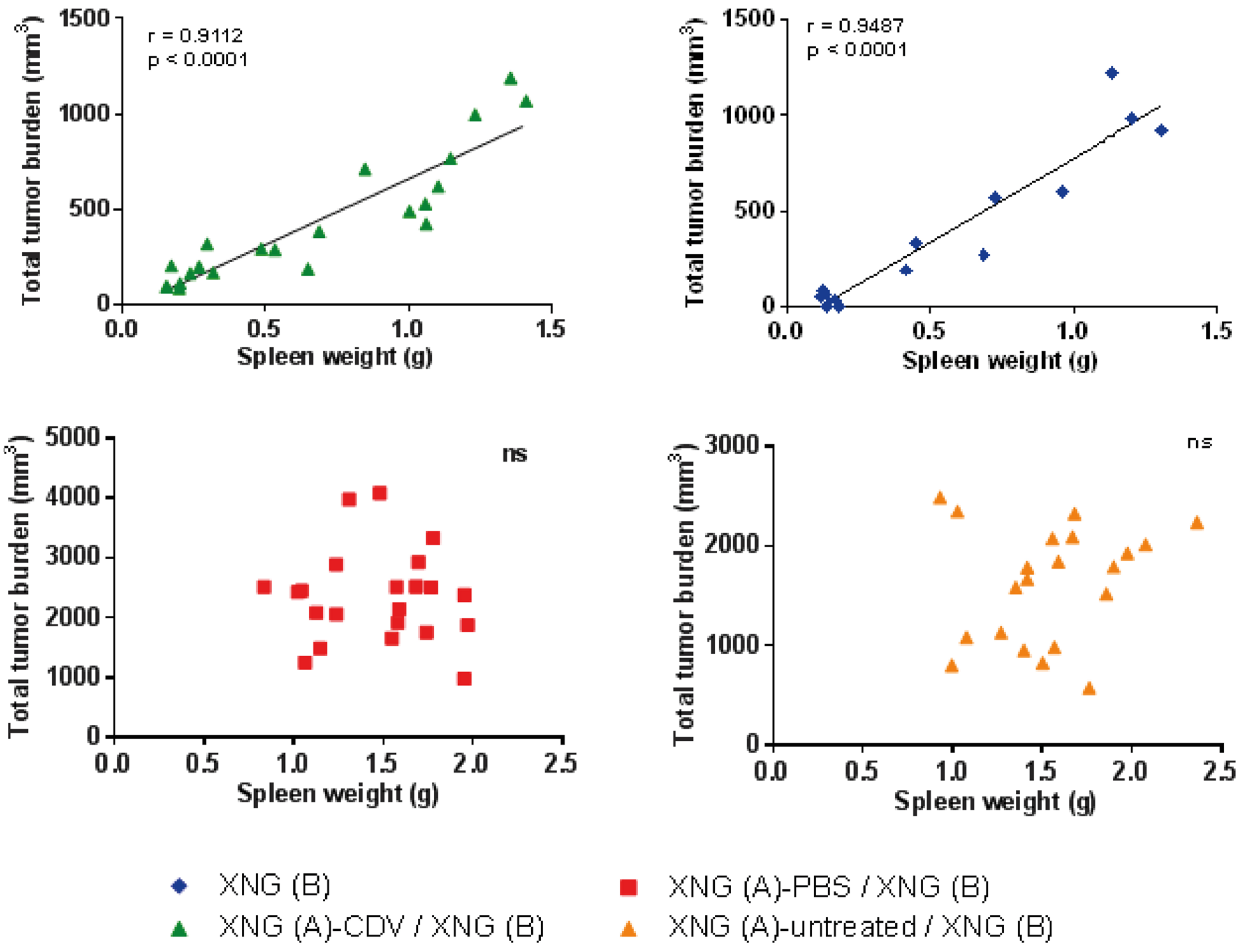

Supplementary Figure 2: Pearson correlation of spleen weight (g) *versus* the sum of XNG (A) and XNG (B) volume (mm^3) for each group individually.

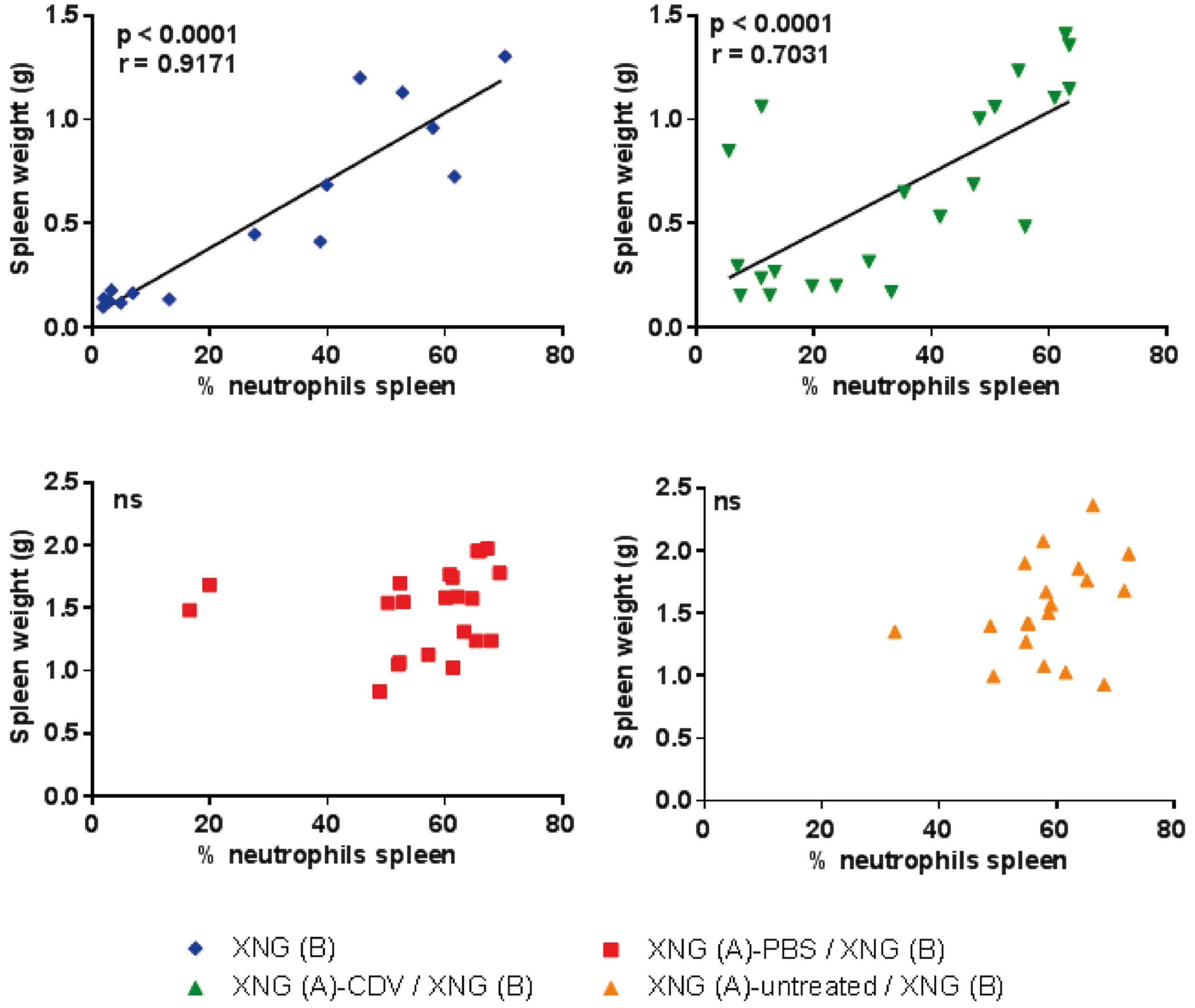

Supplementary Figure 3: Pearson correlation between spleen weight (g) and the percentage of neutrophils in spleen for each group individually.

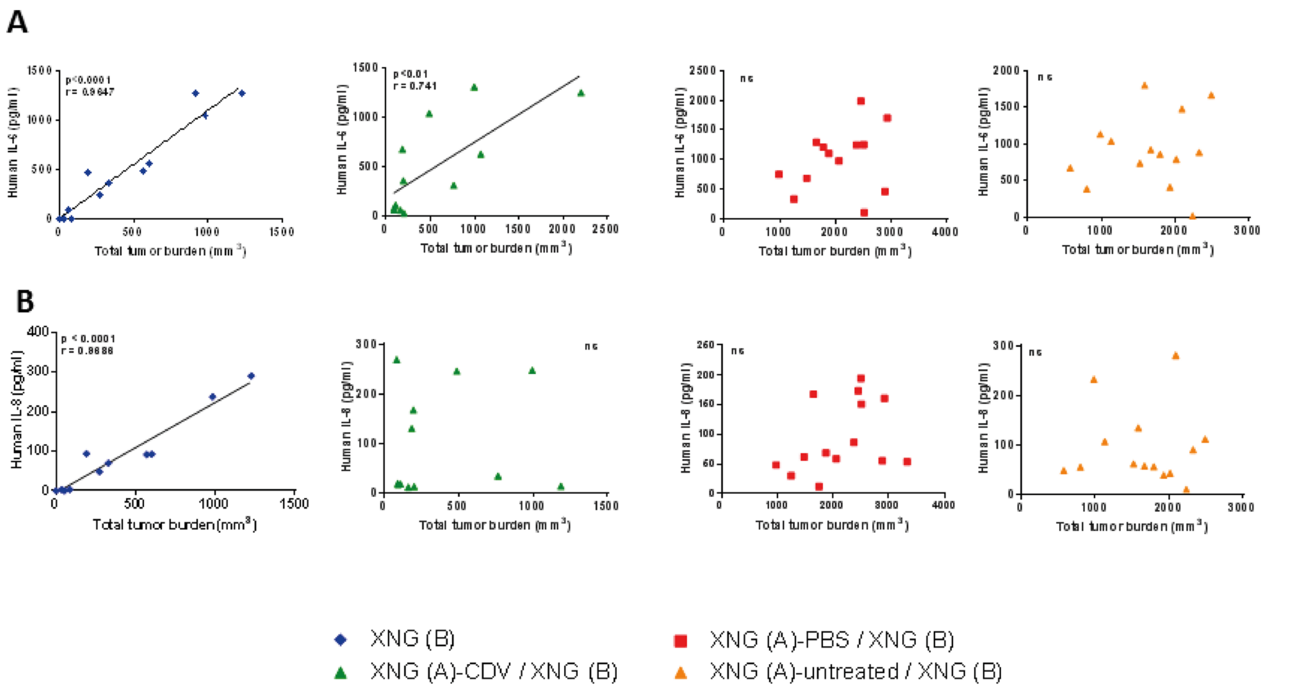

Supplementary Figure 4: Pearson correlation between human IL-6 **(A)** or human IL-8 **(B)** and the total tumor burden (mm^3) for each group individually.

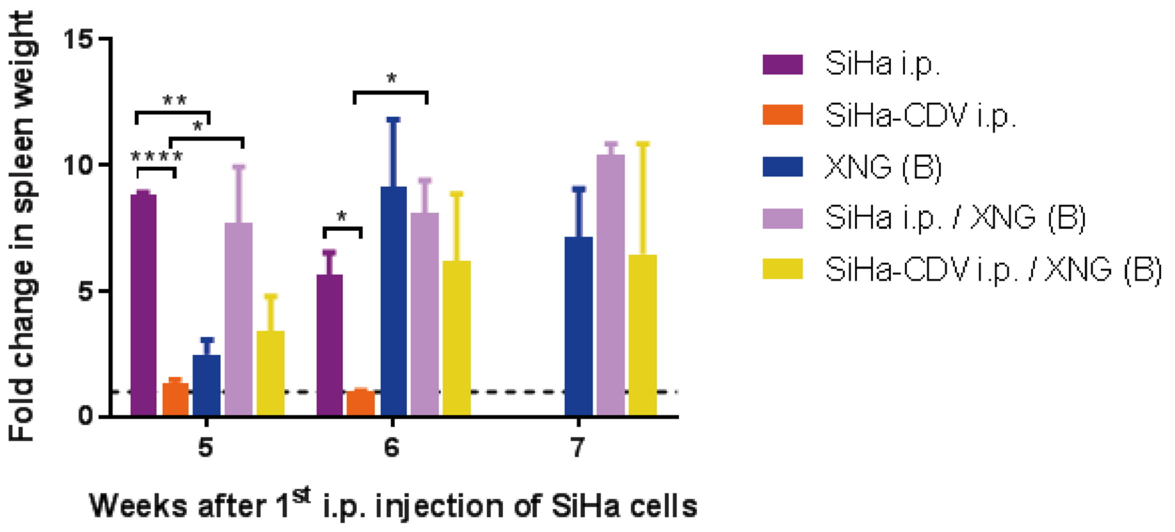

Supplementary Figure 5: Development of splenomegaly in the intraperitoneal-subcutaneous double xenograft mouse model. Fold change in spleen weight compared to control healthy mice, which had an average spleen weight of 0.1 g. Weight of the spleens was determined immediately after dissection of the mice and is shown as mean ± SEM(N=2-3). Statistical significance was indicated as follows: p<0.05 (*); p<0.01 (**); p<0.001 (***); p<0.0001 (****).

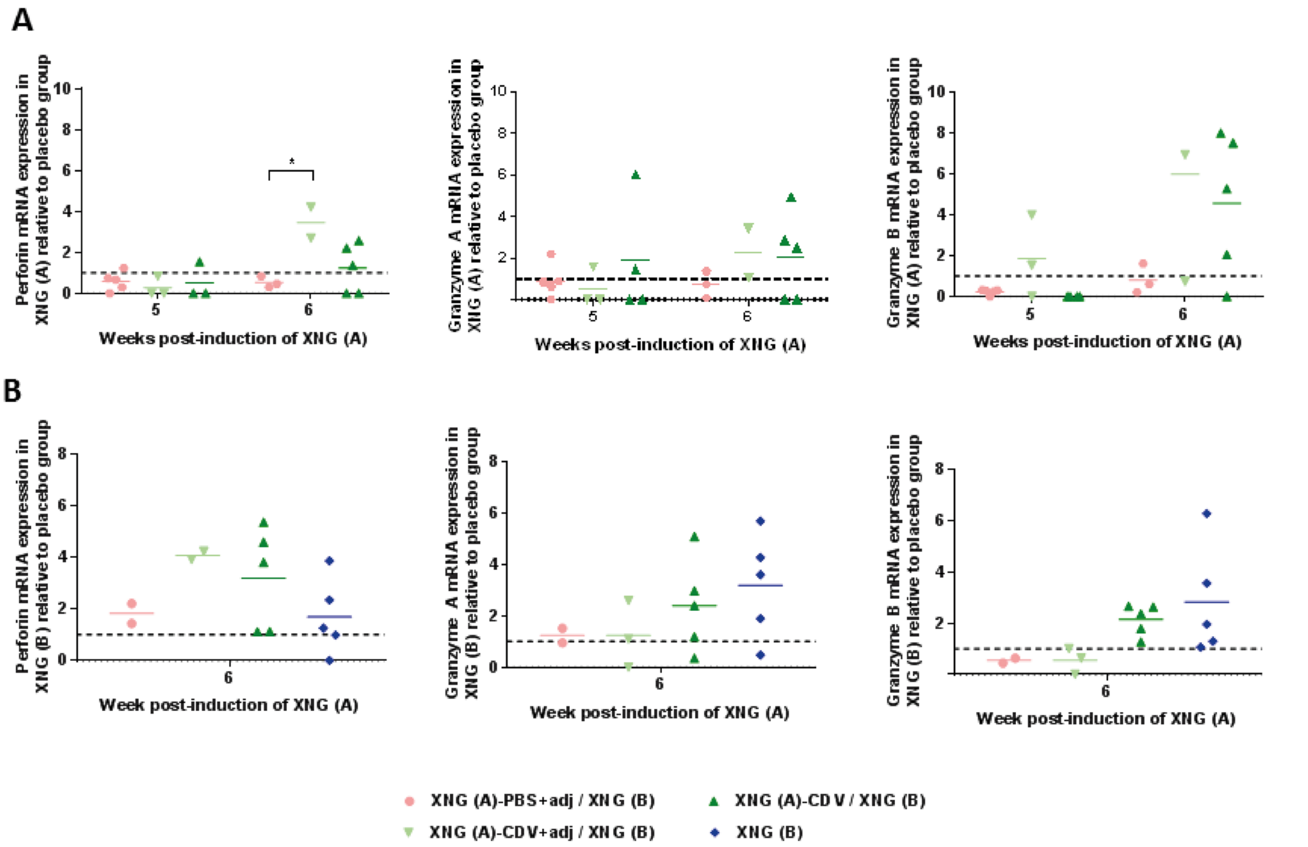

Supplementary Figure 6: Effect of adjuvant treatment (aluminium hydroxide and MPL) on perforin and granzymes mRNA expression in primary **[XNG (A)]** and secondary **[XNG (B)]** subcutaneous SiHa cells xenografts. Relative quantification of perforin and granzymes A and B mRNA expression in XNG (A) **(A)** and XNG (B) **(B)** (N = 2-5) relative to placebo. GAPDH was used as housekeeping gene and all analyses were performed using GAPDH normalization. Relative gene expression was calculated using the $2^{-\Delta\Delta Ct}$ method. $p<0.05$ (*); $p<0.01$ (**); $p<0.001$ (***); $p<0.0001$ (****).

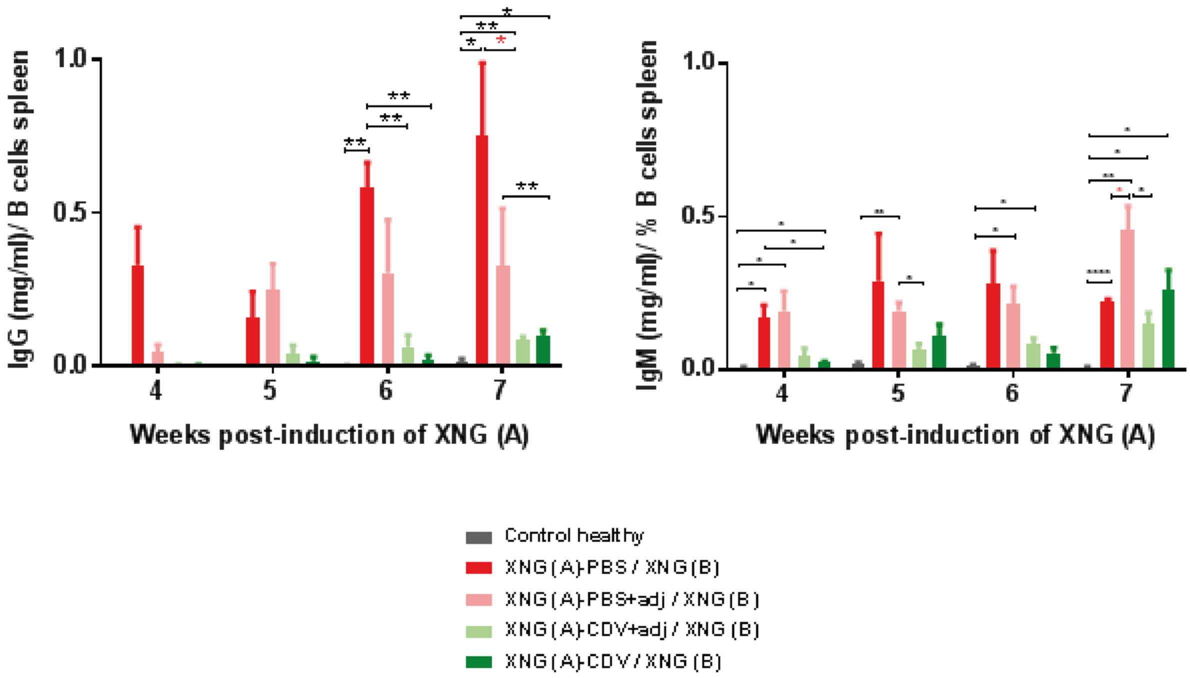

Supplementary Figure 7: Effect of adjuvant treatment (aluminum hydroxide and MPL) on immunoglobulin levels in the sera of mice bearing double SiHa cells xenografts. Ratio of serum IgG and IgM levels on splenic B cells. Immunoglobulin levels were determined in the serum by means of ELISA. Results are shown as fold change of 3-5 values. $p<0.05$ (*); $p<0.01$ (**); $p<0.001$ (***); $p<0.0001$ (****).
